# High temperature induced changes in quality and yield parameters of tomato (*Solanum lycopersicum* L.) and similarity coefficients among genotypes using SSR markers

**DOI:** 10.1016/j.heliyon.2021.e05988

**Published:** 2021-02-03

**Authors:** Amrutha Vijayakumar, Shanija Shaji, R. Beena, S. Sarada, T. Sajitha Rani, Roy Stephen, R.V. Manju, M.M. Viji

**Affiliations:** Department of Plant Physiology, College of Agriculture, Kerala Agricultural University, Vellayani, Thiruvananthapuram, India

**Keywords:** High temperature stress, Tomato, SSR markers, Quality traits, Yield traits

## Abstract

High temperature induced by climatic fluctuations are an important threat for plant growth, development and quality of agricultural produces. Adaptableness to environmental changes generally derives from a large set of genetic traits affecting physio-morphological, biochemical and agronomic parameters. Therefore, the identification of genotypes with higher yield and good quality parameters at high temperatures is becoming increasingly necessary for future breeding programs. Here, we analyzed the performance of different tomato genotypes grown under elevated temperatures in terms of yield and nutritional quality of the fruit. High temperature stress was induced from flower initiation to maturity stage by keeping the pots in a temperature controlled green house facility for 45 days. The quality and yield parameters were taken at the harvesting stage. Starch and soluble sugar concentration in the leaves of tomato genotypes showed significant reduction in its amount under heat stress. Titrable acidity (TA), total soluble solids (TSS) and ascorbic acid content of tomato fruits were highest under high temperature conditions compared to ambient condition but lycopene content decreased with rise in temperature. The yield attributes *viz*., number of fruits/plant, fruit set %, average fruit weight (g), yield per plant (g/plant) were significantly lower for Arka Saurabh, Arka Rakshak and Pusa Rohini when compared to other genotypes under study. Molecular characterization of selected 22 tomato genotypes were assessed using 25 simple sequence repeat (SSR) markers. Phylogenetic tree was constructed by the unweighted neighbour-joining method (UPGMA) using NTSYSpc cluster analysis software. The Jaccard's similarity matrix was constructed using the SIMQUAL method using UPGMA algorithm in NTSYSpc. Jaccard's similarity matrix among these tomato genotypes ranged from a minimum of 0.22 to a maximum of 1 with an average genetic similarity of 0.67. Hence this study has importance in identifying genotypes that could maintain good quality and higher yield under high temperature condition.

## 1 Introduction

High temperature is one of the major abiotic stress affecting plants, having adverse effects on both growth and reproduction (Beena, 2013). Its impact on agriculture is severe by affecting the productivity of crop negatively. The global average temperature has increased by 0.6 °C over the past 100 years and is projected to increase at a rapid rate in future (Root et al., 2003). The average increase is expected to be 0.5–2.8 °C by the end of the 21^st^ century (Meehl et al., 2005; Vuuren et al., 2008; Beena et al., 2018b). Tomato (*Solanum lycopersicum* L.) is considered as an important vegetable crop native to South America. The genus *Solanum* includes annual or short-lived perennial herbaceous plants. It is a typical day neutral plant and is mostly self-pollinated crop. It is an excellent source of carotenoids, vitamins, antioxidants, lycopene and lutein. The limited caloric supply, relatively high fibre content and presence of minerals, vitamins and phenols such as flavonoids make the tomato fruit an excellent ‘‘functional food’’ providing many physiological benefits and basic nutritional requirements. The recent scenario of global warming affected agricultural production and productivity (Ainsworth and Ort, 2010) and the most essential goal of plant breeders should be to develop high yielding varieties that are resistant to biotic and abiotic stress factors. Studies are conducted to evaluate the performance of tomato cultivars for heat tolerance at reproductive phase. Under high temperature condition, reproductive phase is particularly sensitive to continuous mild heat (CMH; Kinet and Peet, 1997). Male sterility and the position of the stigma relative to the anther cone seem to be major factor limiting fruit and seed set (Dane et al., 1991; Levy et al., 1978). Tolerant genotypes were identified under high temperature condition (Levy et al., 1978; Dane et al., 1991; Sato et al., 2000, 2004; Bhattarai et al., 2016). High temperature stress changes the physiological and biochemical responses in plants (Camejo et al., 2005; Min et al., 2014) which later on decreases crop quality and its yield. However, the susceptibility of plants to high temperature differs according to genotypes and also the developmental stages (Wahid et al., 2007). Variation in the response of cultivars to high temperature stress is not only in the vegetative organs (Camejo et al., 2006) but also in the reproductive organs (Firon et al., 2006). The relationship between the reproductive stage of tomato and the average daily temperature was studied and found that the number of fruits, fruit set percentage and fruit weight per plant decreased with increase in daily temperatures of 25–29 °C (Harel et al., 2014). At high temperature, plants transpire more, and yield is reduced because of the impaired pollen, anther development, and reduced pollen viability. Temperatures higher than 35 °C reduce the fruit set and delay the development of normal fruit colours (Sato et al., 2006).

Genetic diversity analysis exclusively based on phenotypic traits may not be a reliable measure of genetic differences as they are influenced by environmental factors (Shehzad et al., 2009). Thus, DNA based molecular markers such as RAPD (Random Amplified Polymorphic DNA), SSR (Simple Sequence repeat), AFLP (Amplified Fragment Length Polymorphism) and SNP (Single Nucleotide Polymorphism) have been routinely used to assess the genetic divergence among the genotypes as they are not influenced by environmental factors. Multi-allelic nature and high polymorphism of SSR markers help to establish the relationship among the individuals even with less number of markers. SSR markers are preferred as they are abundant in the genome, well-distributed throughout the genome, hyper-variable, multi-allelic and co-dominant nature, ease of assaying, highly reproducible and highly informative markers are immensely valuable in studies of variation detection, diversity analysis, phylogeny, population structure, gene mapping and association studies (Beena et al., 2012b; Ditta et al., 2018). The knowledge of the extent of genetic variation, diversity and genetic relationships between genotypes of the crop is vital and foundation for developing an improved cultivar possessing high yield, good fruit quality and adapted to various abiotic and biotic stresses situations (Sheshshayee et al., 2011). Knowledge of the genetic diversity of tomato genotypes is useful for core collection development and effective conservation strategy. The Jaccard's similarity matrix can be constructed using the SIMQUAL method using UPGMA algorithm in NTSYSpc. UPGMA is perhaps the most widely used techniques which is the only method to be suggested if group averages are obtained (Aldenderfer and Blashfield, 1978). Even though the unweighted pair group approach using arithmetic averages (UPGMA) and neighbour-joining (NJ) algorithms is intended to generate single trees, depending mostly on order of data entry, they can derive more than one topology from a single matrix (Stefan Van Dongen and Winnepenninckx, 1996). The UPGMA dendrogram was designed on the basis of similarity indices that demonstrated distinct clustering into groups of different genotypes (Punia et al., 2009). Thus in this study an attempt has been made to (a) evaluate the performance of 22 tomato genotypes for changes in quality and yield traits under high temperature condition. (b) calculate the similarity coefficients among genotypes using SSR markers.

## 2 Materials and methods

### 2.1 Planting material

Planting material used in this study is the cultivated tomato varieties released from various states of India and germplasm collections from Indian Council of Agricultuarl Research-Indian Institute of Horticultural Research, Bangalore (Table 1).

**Table 1:** List of twenty-two tomato genotypes used for the study.

Sl. No.	Varieties	Released from
1	Nandi	UAS & AVRDC
2	IC-45	IIHR collections
3	Pusa Rohini	IARI
4	Pusa Ruby	IARI
5	IIHR-2200	IIHR collections
6	Anagha	KAU
7	Akshaya	KAU
8	Vellayani Vijay	KAU
9	Arka Vikas	IIHR
10	Kashi Vishesh	ICAR-IIVR
11	Vaibhav	UAS & AVRDC
12	IIHR-26372	IIHR collections
13	Palam Pride	HPAU
14	Arka Abha	IIHR
15	Arka Alok	IIHR
16	Manulakshmi	KAU
17	Sakthi	KAU
18	Manuprabha	KAU
19	Arka Samrat	IIHR
20	Arka Sourabh	IIHR
21	PKM-1	TNAU
22	Arka Rakshak	IIHR

UAS - University of Agricultural Sciences, Bangalore.

AVRDC - Asian Vegetable Research and Development Center.

ICAR-IIHR - Indian Institute of Horticultural Research, Bangalore.

ICAR-IARI – Indian Agricultural Research Institute, New Delhi.

KAU – Kerala Agricultural University, Thrissur.

ICAR-IIVR – ICAR- Indian Institute of Vegetable Research, UP.

HPAU – Himachal Pradesh Agricultural University, Solan.

TNAU - Tamil Nadu Agricultural University, Coimbatore.

### 2.2 Methodology

This experiment was conducted at College of Agriculture, Vellayani, Kerala Agricultural University, 8.4316° N, 76.9860° E. Tomato seeds were obtained from NBPGR (substation), Thrissur. Tomato seeds were sown in germination tray (60 cells in one tray of size 54 × 35 × 5 (L x W X H) in cm) and filled with potting mixture (coir pith compost and vermicompost @ 2:1 ratio) and labelling was done properly. Irrigation was provided regularly using a rose can. The one month old seedlings were transplanted to pots (30cm height, 20 cm diameter) with potting mixture made from loamy soil of pH of 5.8, sand and cow dung on equal volume by volume basis. Six replications were maintained for a single variety. The plants were grown in natural, outdoor environment conditions in a wired enclosure (32.1/24 ± 1 °C mean day/night temperature, 1350–1550 μmol m^−2^ s^−1^ light intensity, 60–65% relative humidity) until flower initiation. Five plants per each replication were maintained. The experiment was laid out in completely randomized design with two treatment levels i.e. control and high temperature stress (36+/-2 °C) with three replications each. 20 days after transplanting, a set of 22 genotypes with three replicates were transferred to temperature controlled greenhouse for heat stress induction. The average maximum and minimum air temperatures for control condition during crop growth period was 32.1 °C and 24 °C and the average maximum and minimum relative humidity (RH) of air was 90.6% and 59.2% respectively. The daily temperatures including maximum and minimum temperatures were recorded under control as well as heat stress conditions using digital thermo-hygrometer throughout the experiment. Quality parameters and yield parameters were taken at harvesting stage.

### 2.3 Quality parameters

The carbohydrate content in plants was estimated by Phenol-sulphuric acid method (Dubois et al., 2002). The starch content in plant leaves was estimated by Anthrone method (Hodge and Hofreiter, 1962). Glucose content in the sample was calculated using the standard graph.

The lycopene content in the fruit was quantified by the method explained by Rangana (1976). Optical density (OD) of the extract was measured at 503 nm in UV-VIS-spectrophotometer (Elico SL-160) using petroleum ether as a blank. Lycopene content of the sample was calculated by using the following formula:

Absorbance (1 unit) = 3.1206μg lycopene/mL.

(1) $$\text{Lycopene}\left(\text{mg }100{\text{g}}^{-1}\right)=\left(3.1206\times \text{O.D. of sample}\times \text{volume made up}\times \text{dilution}\times 100\right)/\left(\text{weight of sample}\times 1000\right)$$

Titration method was used to estimate titrable acidity (AOAC, 2000). Five tomatoes from each genotype were homogenized in a mixer to a fine puree. Five grams of homogenized tomato puree was extracted with distilled water and made up the volume to 50 mL. Ten mL of filtrate was titrated against 0.01 N NaOH using a drop of phenolphthalein indicator. Acidity was calculated as using citric acid as standard equivalents and expressed as percent of acidity.

(2) Titratable acidity=Volume of NaOH used(mL)/Volume of juice taken(mL)×0.0064×100

TSS in terms of °Brix units was measured in fresh tomato juice using a digital refractometer (Model DG-NXT, ARKO India Ltd).

The ascorbic acid content in plants was estimated volumetrically by the method explained by Sadasivam and Manickam (2008). Working standard solution of 5ml containing 100 μg/ml of ascorbic acid was pipetted out into a 100 ml conical flask. 4% oxalic acid was added to it and titrated against 2, 6- dichlorophenol indophenol dye (V1 mL). End point was noted on appearance of pink colour which persisted for a few minutes. The sample (0.5g) was weighed and ground in a mortar with pestle using 15ml 4% oxalic acid.

The homogenate was filtered through a double layered cheese cloth. The filtrate was made up to a known volume and centrifuged at 10,000 rpm for 10 min. The supernatant was collected and made up to 25ml using oxalic acid. 5.0 ml aliquot was pipetted into a conical flask to which 10ml of 4% oxalic acid was added. This was titrated against dichlorophenol indophenol (DCPIP) solution until the appearance of pink colour (V2 mL). The amount of ascorbic acid is calculated as follows:

The amount of ascorbic acid is calculated as follows:

(3) $$Ascorbic\;acid=\frac{0.5mg}{V_{1}ml}\times \frac{V_{2}}{5ml}\times \frac{100}{weight\;of\;sample}$$

### 2.4 Yield parameters

The height of plants was measured from the base of the stem to the tip of the shoot at harvest stage. and the average height was calculated on per plant basis and expressed in cm.

Fruit set was also expressed in percentage by counting the total number of flowers as well as total number of fruits per plant.

(4) Fruit setting%=(Total number of fruits/Total number of flowers)×100

Average fruit weight was calculated by adding the weight of fruits from each of three replication plants at harvest and divided it by total number of fruits and expressed in grams per fruit.

The weight of all the fruits collected per plant was taken and the total yield was calculated at the harvesting stage.

### 2.5 DNA isolation

Twenty-two genotypes of tomatoes were used for the present study. The leaf samples were obtained from one month old plant samples, and genomic DNA was isolated using CTAB method as defined in the procedure by (Murray and Thompson, 1980). The genomic DNA isolated from 22 varieties of tomatoes were validated using agarose gel electrophoresis.

**Figure 1:**
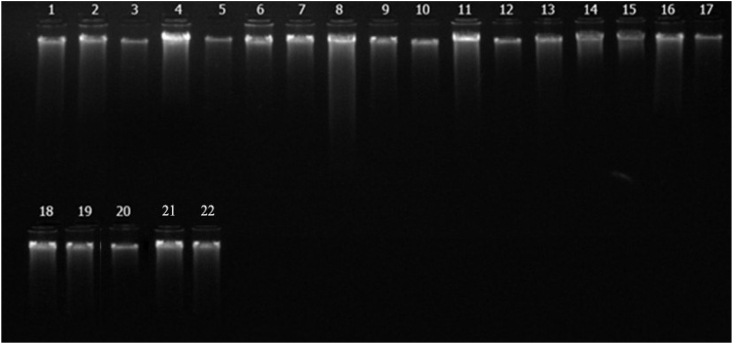
Gel profile with DNA bands of tomato (Lane 1-Manuprabha, Lane 2-Akshaya, Lane 3-Pusa Ruby, Lane 4-IC 45, Lane 5- Nandi, Lane 6-IIHR 2200, lane 7-IIHR 26372, lane 8-Palam Pride, lane 9-PKM 1, lane 10-Manulakshmi, lane 11-Arka Sambrat, lane 12-Rakshak, lane 13-Arka Vikas, lane 14-Pusa Rohini, lane 15-Arka alok, lane 16-Sakthi, lane 17-Vaibhav,lane 18- Vellayani Vijay, lane 19-Anagha, lane 20-Kashi Vishesh, lane 21- Arka Sourabh, lane 22-Arka Abha).

Quality was assessed by using gel electrophoresis with 5μl of crude DNA sample on agarose gel (0.8%) and stained with ethidium bromide. After electrophoresis, the gel was visualized under UV trans-illuminator and photographed with gel documentation system. The observations on the intactness of bands of DNA samples were taken which revealed the quality of the DNA (Figure 1).

### 2.6 PCR amplification using SSR primers

Twenty five SSR primers were randomly selected and tested on isolated genomic DNA of *Solanum lycopersicum* L. (Table 2). These primers are associated with high temperature stress traits and sequences were obtained from the Sol Genomics Network (SGN, http://solgenomics.net/) database. PCR reaction was performed in a 20μl reaction mixture which consisted of, 2.0 μl of genomic DNA with quantity 25ng/μl, 2.0 μl of 10X Taq assay buffer A, dNTPs mix (10mm each) of about 1.5 μl, 0.3 μl of Taq DNA Polymerase (1U). 0.75 μl of Forward and reverse primer (10pM) respectively and 12.7 μl of autoclaved distilled water (Kaushal et al., 2017).

**Table 2:** List of primers along with their sequence, chromosome number and expected product size.

Sl. no.	Chromosome	Primer	Sequence	Expected product size	Reference
1	1	SSR 134	F: CCCTCTTGCCTAAACATCCA	171	Wen et al. (2019)
			R:CGTTGCGAATTCAGATTAGTTG		
2	2	SSR75	F:CCATCTATTATCTTCTCTCCAACAC	155	Wen et al. (2019)
			R:GGTCCCAACTCGGTACACAC		
3	2	SSR 356	F:ACCATCGAGGCTGCATAAAG	259	Wen et al. (2019)
			R:AACCATCCACTGCCTCAATC		
4	2	SSR 605	F:TGGCCGGCTTCTAGAAATAA	196	Wen et al. (2019)
			R:TGAAATCACCCGTGACCTTT		
5	1	SSR 270	F:AGCTCAAGGCTTCTGTTGGA	231	Wen et al. (2019)
			R:AACCACCTCAGGCACTTCAT		
6	2	SSR 96	F:GGGTTATCAATGATGCAATGG	222	Wen et al. (2019)
			R:CCTTTATGTCAGCCGGTGTT		
7	6	SSR 47	F:TCCTCAAGAAATGAAGCTCTGA	191	Wen et al. (2019) and Khan et al. (2020), Kumar et al. (2016)
			R:CCTTGGAGATAACAACCACAA		
8	7	SSR 276	F:CTCCGGCAAGAGTGAACATT	148	Wen et al. (2019)
			R:CGACGGAGTACTTCGCATTT		
9	7	SSR 304	F:TCCTCCGGTTGTTACTCCAC	186	Wen et al. (2019)
			R:TTAGCACTTCCACCGATTCC		
10	8	SSR 63	F:CCACAAACAATTCCATCTCA	250	Wen et al. (2019) and Khan et al. (2020), Kumar et al. (2016)
			R:GCTTCCGCCATACTGATACG		
11	10	SSR 4	F:TTCTTCGGAGACGAAGGGTA	166	Kaushal et al. (2017)
			R: CCTTCAATCCTCCAGATCCA		
12	5	SSR 13	F:GGGTCACATACACTCATACTAAGGA	104	Wen et al. (2019) and Khan et al. (2020), Kumar et al. (2016)
			R:CAAATCGCGACATGTGTAAGA		
13	5	SSR 115	F:CACCCTTTATTCAGATTCCTCT	211	Kumar et al. (2016)
			R:ATTGAGGGTATGCAACAGCC		
14	9	SSR 19	F:CCGTTACCTTGGTCCATCAC	188	Wen et al. (2019) and Khan et al. (2020), Kumar et al. (2016)
			R:GGGAGATGCCACATCACATA		
15	4	SSR 293	F:GCAAAGAGCTCGATCTCCAA	129	Wen et al. (2019)
			R:TTCAGTTACTGGCCTTCGCT		
16	10	SSR 248	F:GCATTCGCTGTAGCTCGTTT	249	Khan et al. (2020), Kumar et al. (2016)
			R: GGGAGCTTCATCATAGTAACG		
17	12	SSR 124	F:TCAATCCATCACACCTTGGA	131	Dhaliwal et al. (2011)
			R: GAGGAAGAAGACCACGCAAA		
18	9	SSR 70	F:TTTAGGGTGTCTGTGGGTCC	120	Dhaliwal et al. (2011)
			R:GGAGTGCGCAGAGGATAGAG		
19	3	SSR 111	F:TTCTTCCCTTCCATCAGTTCT	188	Khan et al. (2020) and Kaushal et al. (2017), Kumar et al. (2016)
			R:TTTGCTGCTATACTGCTGACA		
20	12	SSR 20	F:GAGGACGACAACAACAACGA	157	Khan et al. (2020) and Kaushal et al. (2017), Kumar et al. (2016)
			R:GACATGCCACTTAGATCCACAA		
21	5	SSR 602	F:GGGTCACATACACTCATACTAAGGA	299	Kwon et al. (2009)
			R:GGCAATCATAGCCACTTGGT		
22	4	SSR 450	F:AATGAAGAACCATTCCGCAC	265	Kwon et al. (2009)
			R:ACATGAGCCCAATGAACCTC		
23	1	SSR 341	F:TTCTCTGTGGGTGGCAAT	292	Khan et al. (2020)
			R:AAGCCCCGAATCTGGTAGC		
24	2	SSR 331	F:CGCCTATCGATACCACCACT	178	Khan et al. (2020)
			R:ATGATCCGTTGGTTCGC		
25	11	SSR80	F: GGCAAATGTCAAAGGATTGG	180	Benor et al. (2008)
			R: AGGGTCATGTTCTTGATTGTCA		

### 2.7 Detection of polymorphism among tomato genotypes using SSR primers and analysis of similarity coefficient

Twenty five primer combinations were screened. The documented SSR profiles were carefully examined for the polymorphism in banding pattern among the genotypes. Markers were scored according to the standard protocol using binary codes. Banding patterns were scored for absence (0) and presence (1) of bands.

The amplified gel pictures obtained from twenty five SSR markers were scored. The binary data generated for all the varieties for the polymorphic markers was entered in the NTedit program of NTSYSpc version 2.10 software (Jamshidi and Jamshidi, 2011).

### 2.8 Statistical analysis

The overall effects of treatment and cultivar and their interaction were analysed by means of two-way ANOVA with heat treatment and genotypes taken as fixed factors. Genotypes were treated as fixed factors because we were interested in the response of the specific genotypes used in this experiment. Twenty two varieties were analysed with 3 replications each for the treatment levels. The statistical analysis were done using OPSTAT software (Two factorial CRD).

## 3 Result

Significant genotypic differences for starch content was observed among tomato varieties under high temperature. Among the genotypes, Vaibhav (312.97 mg g^−1^ fresh weight) recorded the maximum starch accumulation followed by Manulakshmi (304.45 mg g^−1^ fresh weight) under control condition, while the minimum starch content was recorded in Arka Rakshak (214.06 mg g^−1^ fresh weight). In heat stress condition, the highest starch content was observed in Anagha (235.67 mg g^−1^ fresh weight), while the lowest was observed in Arka Sourabh (84.37 mg g^−1^ fresh weight). The percent decrease in starch content was more in Arka Sourabh and less in IIHR-2200. The average starch content of the tomato genotypes at flowering stage was 170.71 mg g^−1^ fresh weight and 262.86 mg g^−1^ fresh weight under heat stress and control condition respectively (Table 3).

**Table 3:** Effect of high temperature on starch content in leaves of tomato varieties expressed in mg g^−1^ fresh weight.

Sl. No.	Genotypes	Control	Treatment	Mean
1	Nandi	281.33	222.76	252.04
2	IC-45	250.06	208.07	229.06
3	Pusa Rohini	245.63	114.08	179.85
4	Pusa Ruby	252.98	212.82	232.90
5	IIHR-2200	250.73	127.44	189.09
6	Anagha	271.17	235.67	253.42
7	Akshaya	271.11	211.80	241.46
8	Vellayani Vijay	288.52	191.91	240.22
9	Arka Vikas	209.70	92.33	151.02
10	Kashi Vishesh	279.95	204.87	242.41
11	Vaibhav	312.97	200.21	256.59
12	IIHR-26372	239.47	168.27	203.87
13	Palam Pride	264.15	189.25	226.70
14	Arka Abha	221.26	165.21	193.23
15	Arka Alok	280.82	171.93	226.38
16	Manulakshmi	304.45	191.52	247.99
17	Sakthi	245.40	162.70	204.05
18	Manuprabha	283.79	219.24	251.52
19	Arka Samrat	283.12	183.69	233.41
20	Arka Sourabh	262.54	84.37	173.46
21	PKM-1	269.61	103.21	186.41
22	Arka Rakshak	214.06	94.20	154.13
	Mean	262.86	170.71	
	Factors	SE(m)	C.D. (0.5%)	
	Varieties	3.65	10.29	
	Treatments	1.10	3.10	
	Factor (V X T)	5.17	14.55	

Significant genotypic differences for soluble sugar content was observed in different genotypes under high temperature. Highest soluble sugar concentration was observed in Nandi (77.73 mg g^−1^ fresh weight) and lowest concentration in Arka Rakshak (51.92 mg g^−1^ fresh weight) under control condition, whereas under stress condition Vellayani Vijay (59.6 mg g^−1^ fresh weight) showed the highest soluble sugar content and minimum in Arka Rakshak (35.73 mg g^−1^ fresh weight) Table 4. The average soluble sugar content of the tomato genotypes at flowering stage was 48.83 mg g^−1^ fresh weight and 61.19 mg g^−1^ fresh weight under heat stress and control conditions respectively. The percent decrease in starch content was more in Arka Rakshak and less in Arka Abha.

**Table 4:** Effect of high temperature on soluble sugar content in leaves of tomato varieties expressed in mg g^−1^ fresh weight.

Sl. No.	Genotypes	Control	Treatment	Mean
1	Nandi	77.73	53.65	65.69
2	IC-45	57.52	41.54	49.53
3	Pusa Rohini	56.03	41.35	48.69
4	Pusa Ruby	60.65	49.38	55.02
5	IIHR-2200	57.23	55.53	56.38
6	Anagha	66.43	49.74	58.09
7	Akshaya	65.88	56.82	61.35
8	Vellayani Vijay	67.79	59.60	63.70
9	Arka Vikas	53.41	46.41	49.91
10	Kashi Vishesh	65.99	53.30	59.65
11	Vaibhav	63.67	51.06	57.36
12	IIHR-26372	54.58	42.33	48.46
13	Palam Pride	61.80	42.80	52.30
14	Arka Abha	55.91	50.07	52.99
15	Arka Alok	57.40	53.46	55.43
16	Manulakshmi	72.03	56.81	64.42
17	Sakthi	56.96	45.80	51.38
18	Manuprabha	71.83	52.91	62.37
19	Arka Samrat	62.56	43.98	53.27
20	Arka Sourabh	54.94	46.30	50.62
21	PKM-1	53.83	45.61	49.72
22	Arka Rakshak	51.92	35.73	43.83
	**Mean**	61.19	48.83	
	**Factors**	**SE(m)**	**C.D. (0.5%)**	
	**Varieties**	0.976	2.748	
	**Treatments**	0.294	0.829	
	**Factor (V X T)**	1.380	3.886	

**Figure 2:**
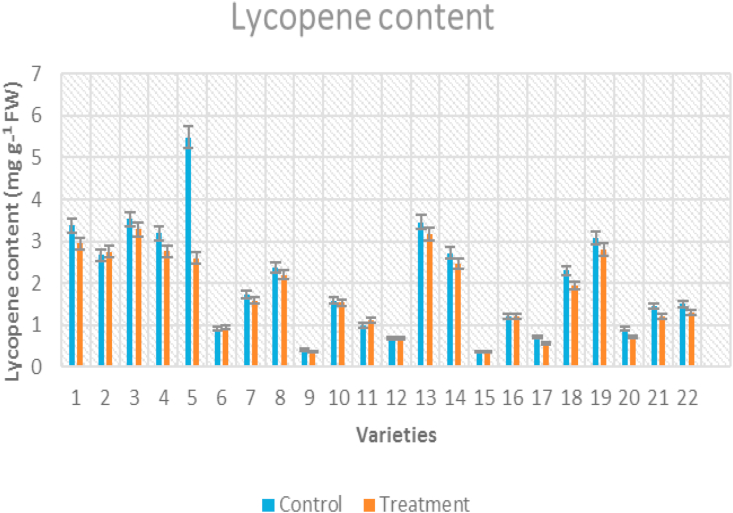
Clustered column graph showing lycopene content of tomato genotypes maintained at control and high temperature conditions.

The lycopene content decreased with a rise in temperature and ambient condition recorded the highest lycopene content in fruits (2.03 mg g^−1^ fresh weight). The highest lycopene content was recorded in IIHR-2200 (5.49 mg g^−1^ fresh weight) and the lowest was observed in Arka Alok (0.36 mg g^−1^ fresh weight) under the control conditions whereas, maximum lycopene content was recorded for Nandi (2.94 mg g^−1^ fresh weight) and minimum was recorded for Arka Vikas (0.35 mg g^−1^ fresh weight) under high temperature conditions (Figure 2). The percent reduction in lycopene content under stress condition was maximum for IIHR-2200 (52%) and minimum for Kashi Vishesh (3%).

**Figure 3:**
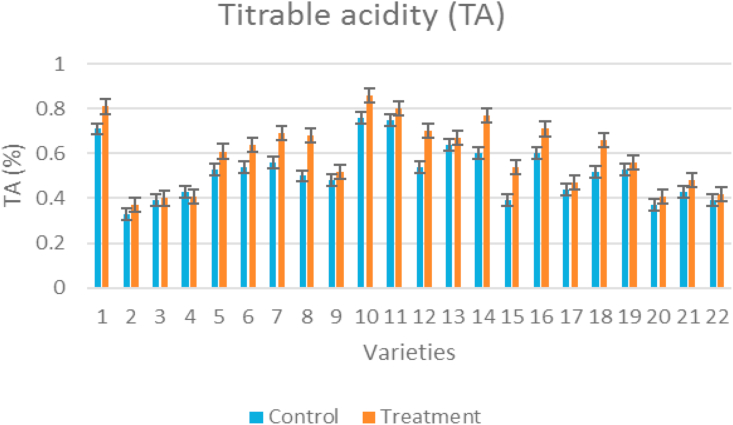
Clustered column graph showing titrable acidity of tomato genotypes maintained at control and high temperature conditions.

Titratable acidity of tomato fruits was found to be significantly different among different genotypes. Highest concentration recorded at high temperature condition when compared to low temperature regimes (control). The highest titrable acidity was recorded for Kashi Vishesh (0.76%) which is at par with Vaibhav (0.75%) and Nandi (0.71%), minimum was recorded for IC-45 (0.33%) under control condition and maximum for Kashi Vishesh (0.86%) which is at par with Vaibhav (0.80%) and Nandi (0.81%), and minimum for IC-45 (0.37%) under high temperature condition (Figure 3). The average titrable acidity under control condition was 0.52% and 0.60% under high temperature condition. The percent increase in titrable acidity under heat stress was highest for Arka Alok (27%) and minimum for Pusa Rohini (2%).

**Figure 4:**
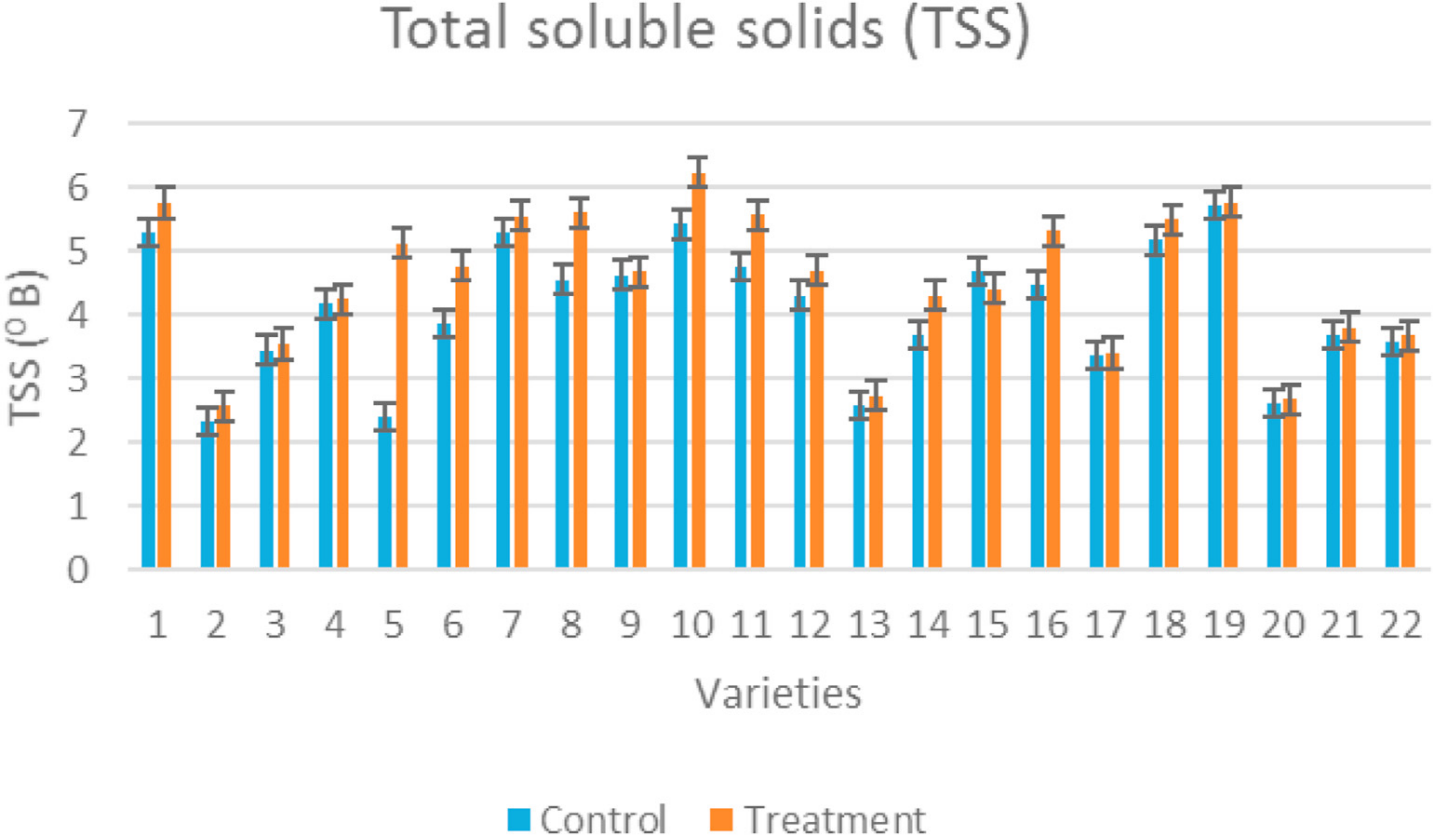
Clustered column graph showing total soluble solids of tomato genotypes maintained at control and high temperature conditions.

In our study, TSS increased in all the genotypes under temperature stress condition compared to control (Figure 4). Highest TSS was recorded for Arka Samrat (5.72%) and lowest for IC-45 (2.32%) under control ambient condition. But under high temperature condition highest TSS was recorded for Kashi Vishesh (6.23%) and lowest for IC-45 (2.57%). The percent increase in TSS was highest for IIHR-2200 (53%) and lowest for Arka Vikas (1%) under stress condition.

**Figure 5:**
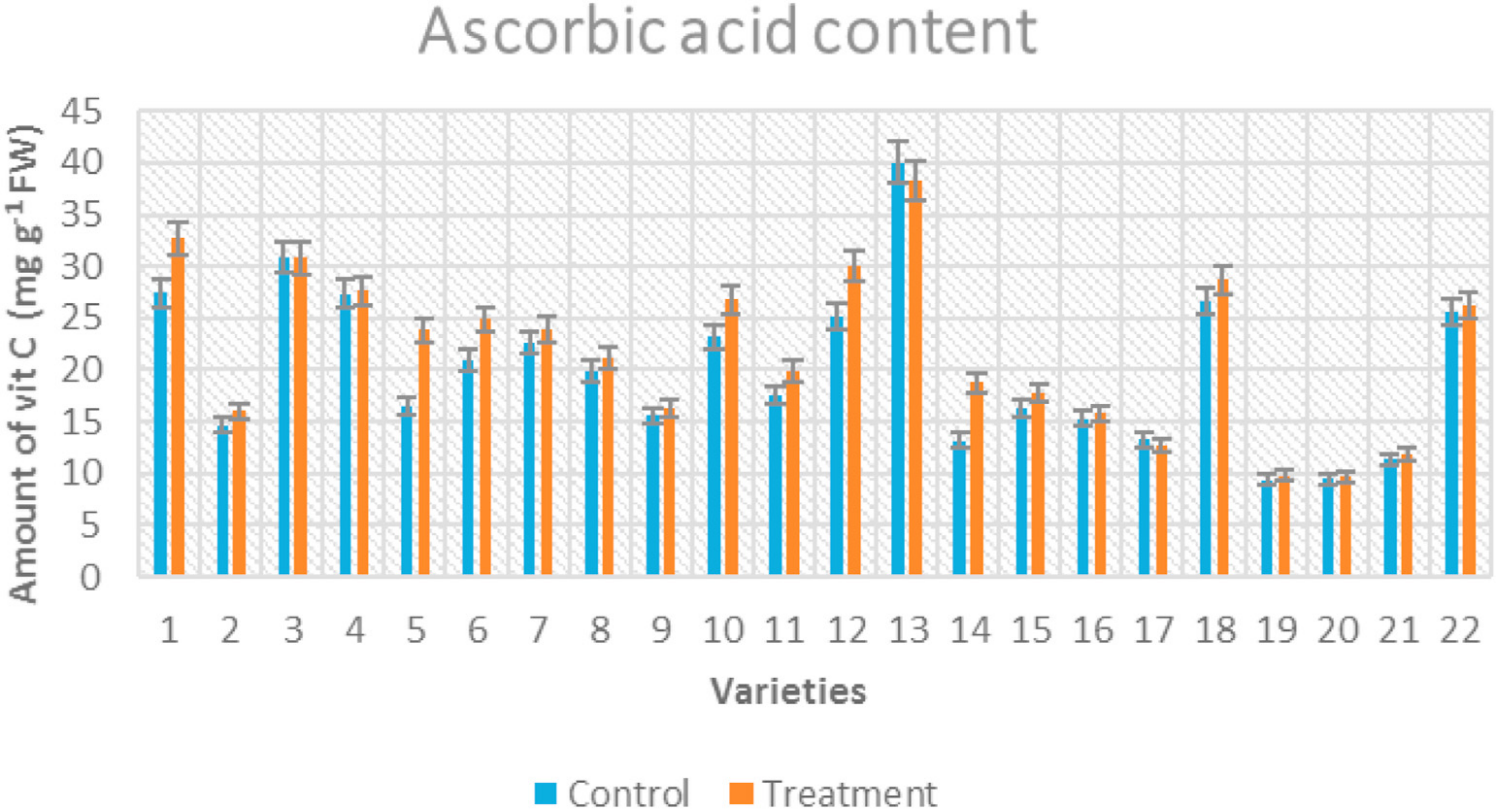
Clustered column graph showing ascorbic acid content of tomato genotypes maintained at control and high temperature conditions.

Vitamin C content showed significant differences among the genotypes, all tolerant genotypes showed higher vitamin C under temperature stress conditions compared to control (Figure 5). Under high temperature condition, highest concentration of vitamin C was observed for Nandi (32.71 mg g^−1^ fresh weight) and lowest for Arka Sourabh (9.67 mg g^−1^ fresh weight) whereas, ascorbic acid was found highest in Palam Pride (40 mg g^−1^ fresh weight) and lowest in Arka Samrat (9.39 mg g^−1^ fresh weight) for control conditions. The percent increase in vitamin C content was maximum for IIHR-2200 (30%) and minimum for Pusa Ruby (1%).

Under high temperature stress in polyhouse condition, all the genotypes showed an increment in the plant height because of the shading effect of polyhouse (Table 5) and elevated CO_2_ (570 μmol mol^−1^). Maximum plant height was observed for Nandi (143.97 cm) and minimum height for Vellayani Vijay (51.9 cm) under control condition and for high temperature condition, highest value of plant height was observed for IC-45 (219.33 cm) and lowest for Arka Sourabh (128.33 cm). The average value of plant height under control and temperature stress condition were 96.79 cm and 162.21 cm respectively. The percent increase in plant height was maximum for Vellayani Vijay (70%) and minimum for Arka Vikas (14%).

**Table 5:** Effect of high temperature on plant height of tomato expressed in cm.

Varieties	Control	Treatment	Mean
Nandi	143.97	161.33	152.65
IC-45	85.67	219.33	152.50
Pusa Rohini	101.70	154.67	128.18
Pusa Ruby	104.17	172.67	138.42
IIHR-2200	113.00	166.33	139.67
Anagha	96.40	146.67	121.53
Akshaya	101.83	165.33	133.58
Vellayani Vijay	51.90	176.67	114.28
Arka Vikas	127.57	148.67	138.12
Kashi Vishesh	84.33	147.00	115.67
Vaibhav	91.87	167.67	129.77
IIHR-26372	109.97	174.67	142.32
Palam Pride	101.23	164.00	132.62
Arka Abha	93.33	183.00	138.17
Arka Alok	68.83	163.67	116.25
Manulakshmi	80.33	172.67	126.50
Sakthi	90.17	130.33	110.25
Manuprabha	106.37	142.67	124.52
Arka Samrat	96.93	159.67	128.30
Arka Sourabh	73.50	128.33	100.92
PKM-1	109.67	174.33	142.00
Arka Rakshak	96.77	149.00	122.88
**Mean**	96.80	162.21	
**Factors**	**SE(m)**	**C.D. (0.5%)**	
**Varieties**	6.75	19.00	
**Treatments**	2.04	5.73	
**Factor (V X T)**	9.55	26.87	

Fruit set significantly decreased at high temperature in all the tomato genotypes as compared to control temperature (Table 6). Highest fruit set % under control condition was recorded in Vellayani Vijay (53.68%) and lowest in Pusa Rohini (13.56%) whereas, highest fruit set % for high temperature stress condition was recorded in IC-45 (7.69%) and lowest for Palam Pride (1.23%). The average fruit set percentage under control and high temperature stress was 33.52% and 2.87% respectively. The percent decrease in fruit set % was maximum for Palam Pride (96.42%) and minimum for Arka Rakshak (86.17%). Significant decrease was observed in average fruit weight of tomato genotypes at high temperature. There was decrease in number of fruits per plant, percent fruit set and fruit yield per plant in all tomato genotypes under high temperature. Nandi, Kashi Vishesh, Anagha showed lesser magnitude of reduction for parameters like lycopene content and fruit set % as compared toArka Sourabh, Pusa Rohini, PKM-1.

**Table 6:** Effect of high temperature on fruit set percentage of tomato genotypes expressed in %.

Varieties	Control	Treatment	Mean
Nandi	44.55	5.56	25.05
IC-45	30.56	7.69	19.13
Pusa Rohini	13.56	1.59	7.57
Pusa Ruby	32.63	2.38	17.51
IIHR-2200	31.79	2.08	16.94
Anagha	40.66	4.17	22.42
Akshaya	42.81	2.73	22.77
Vellayani Vijay	53.68	2.30	27.99
Arka Vikas	15.27	1.96	8.61
Kashi Vishesh	48.72	5.13	26.92
Vaibhav	38.10	2.38	20.24
IIHR-26372	31.64	2.90	17.27
Palam Pride	34.43	1.23	17.83
Arka Abha	36.33	2.86	19.59
Arka Alok	35.80	3.03	19.42
Manulakshmi	35.82	1.90	18.86
Sakthi	29.43	2.22	15.83
Manuprabha	35.68	2.15	18.41
Arka Samrat	36.70	2.56	19.63
Arka Sourabh	26.16	2.56	14.36
PKM-1	24.60	2.22	13.41
Arka Rakshak	18.54	2.56	10.55
**Mean**	33.52	2.87	
**Factors**	**SE(m)**	**C.D. (0.5%)**	
**Varieties**	3.18	8.96	
**Treatments**	0.96	2.70	
**Factor (V X T)**	4.50	12.67	

Average fruit weight was significantly decreased at high temperature in all the tomato genotypes as compared to control temperature (Table 7). The maximum average fruit weight was observed for Arka Vikas (37.23g) and minimum for IC-45 (3.91g) under control conditions whereas, it is maximum for Kashi Vishesh (6.61g) which is on par with Nandi (6.30g) and minimum for Arka Rakshak, Arka Samrat, Arka Sourabh, PKM-1 (0.12g). The maximum percent decrease under heat stress was recorded for Pusa Rohini, Pusa Ruby, Arka Rakshak, Arka Samrat, Arka Sourabh, PKM-1 (susceptible varieties- > 95%) and minimum for IC-45 (77%) as compared to ambient condition.

**Table 7:** Effect of high temperature on average fruit weight content of tomato genotypes expressed in g.

Varieties	Control	Treatment	Mean
Nandi	26.91	6.30	16.61
IC-45	3.91	0.96	2.43
Pusa Rohini	34.78	0.27	17.53
Pusa Ruby	32.41	0.15	16.28
IIHR-2200	15.00	1.14	8.07
Anagha	19.84	3.46	11.65
Akshaya	23.01	1.45	12.23
Vellayani Vijay	17.08	3.25	10.16
Arka Vikas	37.23	0.14	18.68
Kashi Vishesh	17.24	6.61	11.92
Vaibhav	23.99	1.49	12.74
IIHR-26372	20.86	1.28	11.07
Palam Pride	31.11	0.16	15.64
Arka Abha	35.18	1.21	18.19
Arka Alok	16.04	0.97	8.51
Manulakshmi	19.88	1.03	10.46
Sakthi	16.18	0.34	8.26
Manuprabha	29.66	1.02	15.34
Arka Samrat	31.10	0.12	15.61
Arka Sourabh	18.14	0.12	9.13
PKM-1	14.76	0.12	7.44
Arka Rakshak	11.21	0.12	5.66
**Mean**	22.52	1.44	
**Factors**	**SE(m)**	**C.D. (0.5%)**	
**Varieties**	1.91	5.37	
**Treatments**	0.58	1.62	
**Factor (V X T)**	2.69	7.59	

**Figure 6:**
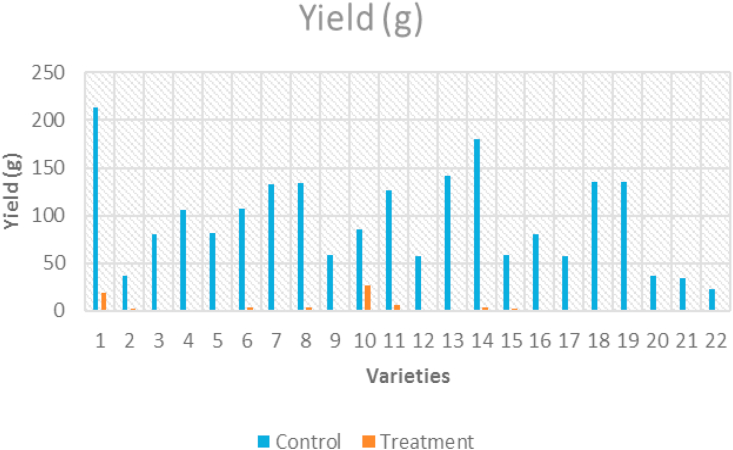
Clustered column graph showing yield obtained from tomato genotypes maintained at control and high temperature conditions.

Yield per plant significantly decreased at high temperature in all tomato genotypes as compared to control temperature. Nandi (213.12g/plant) gave the maximum yield per plant under control condition whereas, Arka Rakshak (22.41g/plant) showed minimum yield per plant (Figure 6). Under heat stress condition, genotypes Nandi, Anagha, Akshaya, IIHR-2200, Vellayani Vijay, Kashi Vishesh, Arka Abha, Arka Alok, Vaibhav, Manuprabha, Manulakshmi, IC-45 and IIHR-26372 recorded higher fruit yield per plant. Varieties like Arka Saurabh, Arka Rakshak, PKM-1, Sakthi, Palam Pride, Arka Samrat recorded the maximum percent reduction in yield per plant (99%) and minimum was recorded in Kashi Vishesh (69%).

### 3.1 Marker analysis

**Figure 7:**
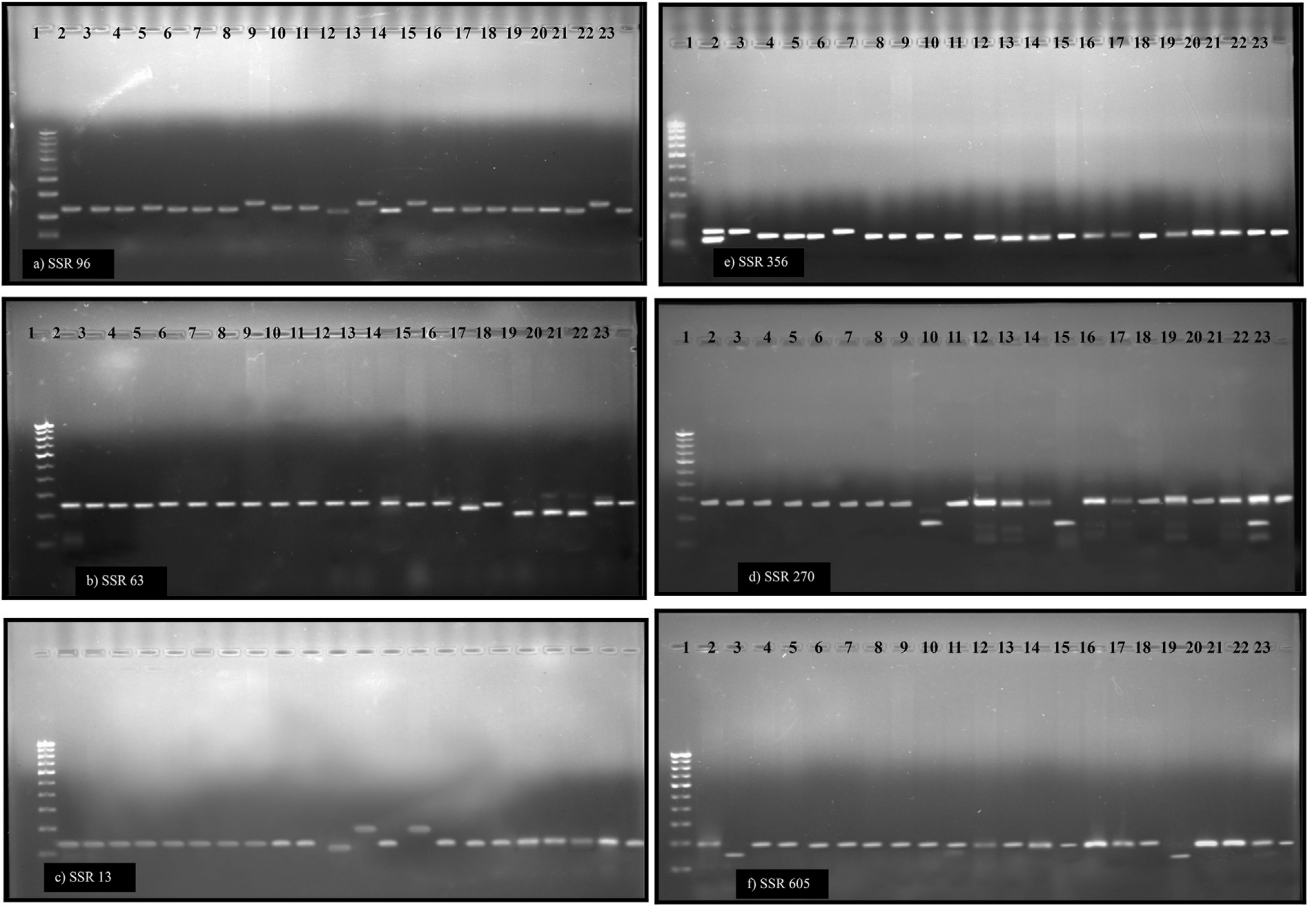
Amplification profile of 22 genotypes with a) SSR 96, b) SSR 63, c) SSR 13, d) SSR 270, e) SSR 356, f) SSR 605. 1-Marker 100 bp ladder. Lane 2–23 tomato genotypes in the same order of Table 1.

Twenty-five SSR markers were used for PCR screening, and the sequence was taken from the Sol Genomics Network database. In 3 percent agarose gel electrophoresis, 7 out of the 25 primers displayed polymorphism (Figure 7) and the other primers were monomorphic. Therefore seven markers were used for determining the coefficient of similarity. The temperatures of these reactions were optimized using gradient PCR technique. Different annealing temperatures (Tm ± 5 °C) were set between each block in this process.

### 3.2 Similarity coefficient analysis

**Figure 8:**
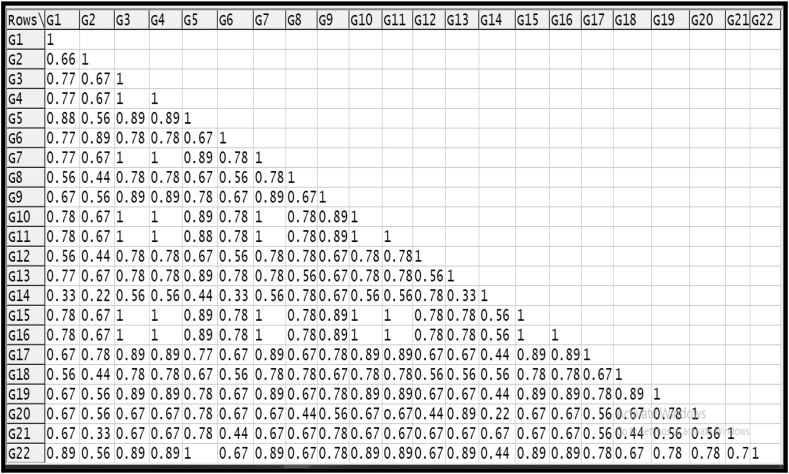
Jaccard's similarity coefficient matrix for 22 tomato genotypes based on SSR data. Where, G1 = Manuprabha, G2 = Akshaya, G3 = Pusa Ruby, G4 = IC-45, G5 = Nandi, G6 = IIHR-2200,G7 = IIHR-26372, G8 = Palam Pride, G9 = PKM-1, G10 = Manulakshmi,G11 = Arka Samrat, G12 = Arka Rakshak, G13 = Arka Vikas, G14 = Pusa Rohini, G15 = Arka Alok, G16 = Sakthi,G17 = Vaibhav, G18 = Vellayani Vijay, G19 = Anagha,G20 = Kashi Vishesh, G21 = Arka Sourabh, G22 = Arka Abha.

Based on the DNA banding pattern of twenty two tomato genotypes using 25 SSR markers, Jaccard's similarity coefficient were developed and displayed in Figure 8. The genetic similarity coefficients of these tomato genotypes ranged from a minimum of 0.22 to maximum of 1. The average genetic similarity range was 0.67.

Maximum genetic similarity (1) was shown by; Pusa Ruby with IC-45, IIHR-26372, Manulakshmi, Arka Samrat, Arka Alok, Sakthi and Arka Abha with Nandi. Quality and yield parameters also showed similarities among these genotypes. Titrable acidity content of Arka Abha and Nandi ranged from 0.6 to 0.7 and for Pusa Ruby with IC-45, IIHR-26372, Manulakshmi, Arka Samrat, Arka Alok, Sakthi ranged from 0.4 to 0.6. Fruit set percentage of Nandi and Arka Abha ranged from 36 to 44% while it ranged from 30 to 36% in case of Pusa Ruby with IC-45, IIHR-26372, Manulakshmi, Arka Samrat, Arka Alok, Sakthi. Yield of Nandi and Arka Abha showed a range between 180-213 g/plant and that of Pusa Ruby with IC-45, IIHR-26372, Manulakshmi, Arka Samrat, Arka Alok, Sakthi ranged 50–80 g/plant.

Minimum genetic similarity coefficient (0.22) was shown by two pairs of genotypes viz. Pusa Rohini with Akshaya and Kashi Vishesh. Since they have low similarities, they shown differences in the yield, physiological data and in molecular characterization. In case of yield, Pusa Rohini with Akshaya and Kashi Vishesh shown wide range differences in lycopene content (1.58–3.53 mg plant^−1^), fruit set percentage (13–48%) and yield (80–135g/plant).

## 4 Discussion

Heat stress reduces the sucrose transport and its accumulation in the leaves of both heat tolerant and heat-sensitive tomato genotypes, indicating that the carbohydrate translocation and partitioning to other plant parts are negatively affected under high temperatures, similar to the results obtained from wheat (Wahid et al., 2007; Shanmugam et al., 2013). A decrease in the starch content was observed under different exposure of abiotic stress (Vinocur and Altman, 2005). In our study also a drastic change in the starch content was observed for different varieties. In heat stress condition, the highest starch content was observed in Anagha, while the lowest was observed in Arka Sourabh. Under heat stress, the concentration of starch and soluble sugar in the pollen grains was lower than that under control conditions (Kumar et al., 2015). These findings are similar to those obtained with that from rice (Sheoran and Saini, 1996) and wheat (Dorion et al., 1996). It has been suggested that carbohydrate starvation in those grains are not responsible for the stress-induced pollen sterility. Pollen of heat tolerant varieties have high amount of glucose rather than sucrose and fructose and it can also retain high amount of carbohydrates (Firon et al., 2006). Xu et al. (2017) revealed that fruit set directly influenced the number of fruits and yield in tomato crop moreover; there is no significant correlation between vegetative and reproductive traits. flower number per inflorescence and membrane thermo-stability are also relevant characteristics and might be used as indicators of reproductive heat tolerance. Several workers also reported that high temperatures cause significant loss in tomato productivity due to reduced fruit set, number of fruits and poor-quality fruit (Zinn et al., 2010; Akhtar et al., 2012; Nahar and Ullah, 2012; Solankey et al., 2017).

Shi and Le Maguer (2000) reported the inhibition of lycopene production at higher temperatures (38 °C). The relatively heat tolerant genotypes showed lesser decrease in lycopene content in the fruit at high temperature as compared to susceptible genotypes (Sharma and Le Maguer, 1996). Lycopene constitutes 80–90 % of the total carotenoids in tomato fruits (Valverde et al., 2002). The result obtained from the present study also pointed the fact that lycopene production under heat stress was severely affected. Under high temperature condition, maximum lycopene content was recorded for Nandi and minimum was recorded for Arka Vikas. Amrutha and Beena (2020) observed variations in tomato genotypes for fruit quality parameters at high temperature conditions. The fruits showed lower content of phenols, flavonoids, ferric reducing antioxidant potential, total soluble solids, and titrable acidity in plants grown at heat stress as compared with the control. The ascorbic acid content was high at stress condition. Carotenoids and lycopene content was low at temperature stress compared to higher content observed at control condition (Mamatha et al., 2014).

Increase in temperature increased TSS and titrable acidity but decreased total sugars, lycopene, and total carotenoids concentration in tomato (Lokesha et al., 2019). The sugars contribute to the total soluble solids content of tomato fruits (Laxman et al., 2013: Selahle et al., 2014). TSS ranged from 4 to 6 °Brix in tomato fruits. The change in the glucose to fructose ratio and the organic acids content is the main cause for changes in the TSS changes in tomato. For the taste of tomatoes, TSS was reported as a beneficial indicator (Klunklin and Savage, 2017). TSS increased in the genotypes under temperature stress compared to control, which is on par with inferences by Shivashankara et al. (2015). Our study also showed that an increment in TA and TSS value were observed for all the tomato genotypes. Under stress conditions Vellayani Vijay showed the highest soluble sugar content and minimum in Arka Rakshak. The phenolic substances have a protective role on ascorbic acid content (Wang and Zheng, 2001) the presence of phenolics and flavonoids in tomato fruits helped to maintain the vitamin C level. A significant increase in total phenolic acids and flavonoids under high temperature were reported in strawberry (Wang, 2006) and also in other crops (Pervez et al., 2009; Bita and Gerats, 2013). Vitamin C content showed significant differences among the tolerant genotypes, all tolerant genotypes showed higher vitamin C under temperature stress conditions compared to control. Vitamin C content increased when the heat stress was imposed during flowering and fruit set stages, indicating that the plant metabolism is adapted to high temperature. The synthesis and accumulation of health-promoting metabolites, termed phytochemicals, depends mainly on the genetic material, although the agronomic practices and environmental factors also have an important influence on yield and quality characteristics of fruits and vegetables (Rouphael et al., 2012; Schreiner et al., 2013). Thus, salt and nutritional stresses have been used for the improvement of the nutritional quality of fruits (Colla et al., 2013; Fanciullino et al., 2014; Massaretto et al., 2018). Heat stress increased abscisic acid (ABA) and reduced salicylic acid (SA) content; however, combined application of SA + HA markedly reduced ABA and increased SA. Antioxidant enzymes activities revealed that SA and HA treated plants exhibited increased levels of ascorbate peroxidase (APX), superoxide dismutase (SOD), and reduced glutathione (GSH) (Hemmati et al., 2015; Gururani et al., 2015). Seed filling parameters recorded at milky and dough stage revealed that high temperature stress condition increased the amount of reducing sugar, carbohydrates, starch, and flavonoids. However, amylose, seed protein, and anthocyanin showed reduction under high temperature stress condition. Activity of invertase was reduced under high temperature condition compared to control in all varieties from 15 to 30 days after 50% flowering (Pravallika et al., 2020).

Fruit setting percentage is affected by changing temperatures during different crop growing seasons (Nahar and Ullah, 2012; Beena et al., 2018a). High temperatures cause significant loss in tomato productivity due to reduced fruit set and poor fruit quality (Mitcham and McDonald, 1992; Khanal, 2012). While shading increased the number of fruits per plant and total fruit yield. The maximum fruit yield was obtained by plants grown under 50% shading in both cultivars under study. Tomato plants grown under shading gave the best physical characteristics of tomato fruits (fruit length and diameter) and TSS %. Leaf concentrations of N, K and Ca were significantly increased with the increased shading levels. The highest content of N, K and Ca was observed with shading with black net at 50% density. On the contrary, plants grown without shading had the highest content of P (El- Bassiony et al., 2014). Alsamir et al. (2017) reported that high temperature in tomato reduced number of fruits, flower to fruit set ratio and fresh fruit weight. These results are supporting the present inferences from our study. The higher pollen viability, high pollen germination and high soluble sugar content in pollen grains at anthesis may be the reason for better number of fruits per plant, percent fruit set and fruit yield in tolerant genotypes at high temperature. Pollen viability and fertility are reported to be reason for better plant productivity during heat stress. The fruit number, fruit set percentage and fruit weight per plant were decreased with increase in temperature. In the present study, the yield attributes viz., number of fruits/plant, fruit set %, average fruit weight (g), yield per plant (g/plant) were significantly lower for varieties like Arka Saurabh, Arka Rakshak and Pusa Rohini.

Under heat stress conditions Nandi, Anagha, Akshaya, IIHR-2200, Vellayani Vijay, Kashi Vishesh, Arka Abha, Arka Alok, Vaibhav, Manuprabha, Manulakshmi, IC-45 and IIHR-26372 produced higher fruit yield per plant. But the varieties like Arka Saurabh, Arka Rakshak, PKM-1, Sakthi, Palam Pride, Arka Samrat recorded the maximum percent reduction in yield per plant and the minimum was recorded in Kashi Vishesh. At high temperature, plants transpire more, and hence yield reduction is caused by the impaired pollen, anther development, and reduced pollen viability. The temperature values higher than 35 °C reduce the fruit set and delay the development of normal fruit colour (Kang et al., 2002; Sato et al., 2006). Reduced allocation of assimilates under high temperature stress compared with control temperature condition (Singh et al., 2005) and reduced supply of photosynthates and poor production of growth regulators in sink tissues are pointed out to be the reasons for reduced yield related traits (Islam, 2011; Hasanuzzaman et al., 2013). Studies on heat-tolerant tomato genotype demonstrated that high *invertase* activity and increased sucrose import into young tomato fruits contributed to heat tolerance through increasing sink strength and sugar signalling activities, by regulating a programmed cell death pathway (Li et al., 2012). The genotypes producing higher proline concentrations in plant parts and with higher membrane thermo-stability under high temperature produced highest fruit yield, and exhibited higher temperature tolerance (Din et al., 2015). Heat tolerant genotypes maintained higher net photosynthesis (*P*_N_) and increased stomatal conductance (*g*_s_) at 38 °C, and better leaf cooling. Sensitive genotypes had lower *F*_v_/*F*_m_ and *P*_N_ at 38 °C, and *g*_s_ increased less than in the tolerant group and less leaf cooling. Under controlled conditions, all eight genotypes had the same plant size and pollen viability, but after heat stress, plant size and pollen viability reduced dramatically in the sensitive group (Gerganova et al., 2019). Two tolerant and two sensitive genotypes were grown in the field during a heat wave (38/26 °C). Tolerant genotypes accumulated more biomass, had a lower heat injury index and higher fruit yield (Poudyal et al., 2019). Expression of the *FeSOD1* gene in transgenic tomato plants under salinity leads to a decrease in the damage of ultrastructure organization of plastids and mitochondria, suggesting a protective effect of this gene under salinity and drought (Baranova et al., 2010; Wang et al., 2016).

### 4.1 Similarity coefficient analysis

A wide range of similarity coefficients between certain genotypes indicated the presence of significant genetic variability between some of the investigated genetic stocks. Among other tomato varieties, Dhaliwal et al. (2011) previously reported similar findings of similarity coefficients among the tomato genotypes. The limited range of coefficient of similarity between these genotypes suggested the existence of limited genetic similarities among the analyzed genotype. A similar study was conducted by Kumar et al. (2016) and reported that the genotypes Arka Vikas and 2012TODVAR-2 are 100% similar.

Based on present study, Akshaya, IIHR-2200, Manuprabha are moderately tolerant varieties showed 89% similarity. Pusa Ruby, IC-26372, Manulakshmi, Arka Samrat, Arka Alok, Sakthi showed 100% similarity. Kaushal et al. (2017) reported a maximum of 96% similarity among tomato. High level of similarity (95%) was revealed among 39 tomato (Al-Abadi, 2007), similarity of 100% was found among tomato (Tam et al., 2005). Kashi Vishesh, Anagha and Vellayani Vijay are tolerant varieties. Kashi Vishesh and Anagha showed 78% similarity. Kashi Vishesh and Vellayani Vijay showed 67% similarity. Vellayani Vijay and Anagha showed 89% similarity. Susceptible varieties are; Arka Sourabh, Pusa Rohini, Palam Pride, Arka Rakshak and observed 78% similarity for three pairs of genotypes viz. Arka Sourabh with Pusa Rohini, Palam Pride and Arka Rakshak. A 68% similarity observed for two pairs of genotypes viz. Pusa Rohini with Palam Pride and Arka Rakshak.

## 5 Conclusion

Significant genotypic differences for starch, soluble sugars, titrable acidity (TA), total soluble solids (TSS), lycopene content, yield attributes *viz*., number of fruits/plant, fruit set %, average fruit weight (g) and yield per plant (g/plant) were observed among tomato genotypes. Nandi, Anagha, Akshaya, Vellayani Vijay, Kashi Vishesh showed high temperature tolerance. Jaccard's similarity coefficient matrix of these tomato genotypes ranged from a minimum of 0.22 to a maximum of 1. Further study has to be conducted for the confirmation of heat tolerance with respect to different attributes contributing for tolerance mechanisms.

## Declarations

### Author contribution statement

Amrutha Vijayakumar: Conceived and designed the experiments; Performed the experiments; Analyzed and interpreted the data; Wrote the paper.

Shanija Shaji; Sarada, S.: Conceived and designed the experiments; Performed the experiments; Analyzed and interpreted the data; Contributed reagents, materials, analysis tools or data; Wrote the paper.

Beena R.: Conceived and designed the experiments; Analyzed and interpreted the data; Contributed reagents, materials, analysis tools or data; Wrote the paper.

Sajitha Rani, T; Roy Stephen.; Manju, R.V.; Viji, M.M.: Contributed reagents, materials, analysis tools or data.

### Funding statement

Acknowledgement to 10.13039/501100009937Kerala Agricultural University for providing student research fund.

### Data availability statement

Data included in article/supplementary material/referenced in article.

### Declaration of interests statement

The authors declare no conflict of interest.

### Additional information

No additional information is available for this paper.

## Appendix A Supplementary data

Gel Pictures Gel Pictures

## References

[bib1] Ainsworth E.A., Ort D.R. (2010). How do we improve crop production in a warming world?. Plant Physiol..

[bib2] Akhtar S., Ansary S.H., Dutta A.K., Karak C., Hazra P. (2012). Crucial reproductive characters as screening indices for tomato (*Solanum lycopersicum*) under high temperature stress. J. Crop Weed.

[bib3] Al-Abadi B. (2007). Genetic Variations Among Jordanian Tomato Landraces Using Simple Sequence Repeat (SSR) and Performance of Their Seedling to Salinity Stress.

[bib4] Aldenderfer M.S., Blashfield R.K. (1978). Computer programs for performing hierarchical cluster analysis. Appl. Psychol. Meas..

[bib5] Alsamir M., Ahmad N.M., Keitel C., Mahmood T., Trethowan R. (2017). Identification of high-temperature tolerant and agronomically viable tomato (*Solanum lycopersicum*) genotypes from a diverse germplasm collection. Adv. Crop Sci. Technol..

[bib6] Amrutha V., Beena R. (2020). Impact of temperature difference on the physicochemical properties and yield of tomato: a review. Chem. Sci. Rev. Lett..

[bib7] Association of official analytical chemists AOAC (1990). Official Methods of Analysis of the Association of Official Analytical Chemists.

[bib8] Baranova E.N., Serenko E.K., Balachnina T.I., Kosobruhov A.A., Kurenina L.V., Gulevich A.A., Maisuryan A.N. (2010). Activity of the photosynthetic apparatus and antioxidant enzymes in leaves of transgenic Solanum lycopersicum and Nicotiana tabacum plants, with FeSOD1 gene. Russ. Agric. Sci..

[bib9] Beena R., Sheshshayee M.S., Madhura J.N., Prasad T.G., Udayakumar M., Sabu A., Anu A. (2012). Development of SSR markers and genetic variability in physiological traits in Bambara groundnut (Vigna subterranea L. Verdc). Prospects in Bioscience: Addressing the Issues.

[bib10] Beena R., Hemantaranjan A. (2013). Research paradigm and inference of studies on high temperature stress in rice (Oryza sativa L.).

[bib11] Beena R., Vighneswaran V., Sindumole P., Narayankutty M.C., Voleti S.R. (2018). Impact of high temperature stress during reproductive and grain filling stage in rice. Oryza. Int. J. Rice.

[bib12] Beena R., Veena V., Narayankutty M.C. (2018). Evaluation of rice genotypes for acquired thermo-tolerance using Temperature Induction Response technique. Oryza. Int. J. Rice.

[bib13] Benor S., Zhang M., Wang Z., Zhang H. (2008). Assessment of genetic variation in tomato (*Solanum lycopersicum* L.) inbred lines using SSR molecular markers. J. Genet. Genom..

[bib14] Bhattarai U., Sharma A., Das R., Talukdar P. (2016). Genetic analysis of yield and yield-attributing traits for high temperature resistance in tomato. Int. J. Veg. Sci..

[bib15] Bita C.E., Gerats T. (2013). Plant tolerance to high temperature in a changing environment: scientific fundamentals and production of heat stress-tolerant crops Fron. Plant Sci..

[bib16] Camejo D., Jimenez A., Alarcon J.J., Torres W., Gomez J.W., Sevilla F. (2006). Changes in photosynthetic parameters and antioxidant activities following heat-shock treatment in tomato plants. Funct. Plant Biol..

[bib17] Camejo D., Rodriguez P., Morales M.A., Dellamico A., Torrecillas, Alarc J.J. (2005). High temperature effects on photosynthetic activity of two tomato cultivars with different heat susceptibility. J. Plant Physiol..

[bib18] Colla G., Rouphael Y., Cardarelli M., Svecova E., Rea E., Lucini L. (2013). Effects of saline stress on mineral composition, phenolics acids and flavonoids in leaves of artichoke and cardamon genotypes grown in floating system. J. Sci. Food Agric..

[bib19] Dane F., Hunter A.G., Chambliss O.L. (1991). Fruit set, pollen fertility, and combining ability of selected tomato genotypes under high-temperature field conditions. J. Am. Soc. Hortic. Sci..

[bib20] Dhaliwal M.S., Singh M., Singh, Cheema D.S. (2011). Genetic diversity analysis and DNA fingerprinting of elite genetic stocks of tomato using SSR markers. Indian J. Genet. Plant Breed..

[bib21] Din J.U., Khan S.U., Khan A., Qayyum A., Abbasi K.S., Jenks M.A. (2015). Evaluation of potential morpho-physiological and biochemical indicators in selecting heat-tolerant tomato (*Solanum lycopersicum* Mill.) genotypes. Hortic. Environ. Biotechnol..

[bib22] Ditta A., Zhou Z., Cai X., Wang X., Okubazghi K.W., Shehzad M., Xu Y., Hou Y., Sajid Iqbal M., Khan M.K.R., Wang K., Liu F. (2018). Assessment of genetic diversity, population structure, and evolutionary relationship of uncharacterized genes in a novel germplasm collection of diploid and allotetraploid *gossypium* accessions using EST and genomic SSR markers. Int. J. Mol. Sci..

[bib23] Dorion S., Lalonde S., Saini H.S. (1996). Induction of male sterility in wheat by meiotic-stage water deficit is preceded by a decline in invertase activity and changes in carbohydrate metabolism in anthers. Plant Physiol..

[bib24] Dubois M., Hamilton J.K., Rebers P., Smith F. (2002). Calorimetric dubois method for determination of sugar and related substances. Anal. Chem..

[bib25] El- Bassiony A.M., Fawzy Z.F., Riad G.S., Ghoname A.A. (2014). Mitigation of high temperature stress on growth, yield and fruit quality of tomato plants by different shading level. Middle East J. Appl. Sci..

[bib26] Fanciullino A.L., Bidel L.P.R., Urban L. (2014). Carotenoid responses to environmental stimuli: integrating redox and carbon controls into a fruit model. Plant Cell Environ..

[bib27] Firon N., Shaked R., Peet M.M., Pharr D.M., Zamski E., Rosenfeld K., Althan L., Pressman E. (2006). Pollen grains of heat tolerant tomato cultivars retain higher carbohydrate concentration under heat stress conditions. Sci. Hortic..

[bib29] Gerganova M.T., Faik A.K., Velitchkova M.Y. (2019). Acquired tolerance of the photosynthetic apparatus to photoinhibition as a result of growing *Solanum lycopersicum* at moderately higher temperature and light intensity Functional. Plant Biol..

[bib31] Gururani M.A., Mohanta T.K., Bae H. (2015). Current understanding of the interplay between phytohormones and photosynthesis under environmental stress. Int. J. Mol. Sci..

[bib32] Harel D., Fadida H., Slepoy A., Gantz S., Shilo K. (2014). The effect of mean daily temperature and relative humidity on pollen, fruit set and yield of tomato grown in commercial protected cultivation. Agronomy.

[bib33] Hasanuzzaman M., Nahar K., Alam M., Roychowdhury R., Fujita M. (2013). Physiological, biochemical, and molecular mechanisms of heat stress tolerance in plants. Int. J. Mol. Sci..

[bib34] Hemmati H., Gupta D., Basu C., Pandey G.K. (2015). Molecular physiology of heat stress responses in plants. Elucidation of Abiotic Stress Signaling in Plants: Functional Genomics Perspectives.

[bib35] Hodge J.E., Hofreiter B.T., Whistler R.L., BeMiller J.N. (1962). Methods in Carbohydrate Chemistry.

[bib36] Islam M.T. (2011). Effect of temperature on photosynthesis, yield attributes and yield of tomato genotypes. Int. J. Expt. Agric..

[bib37] Jamshidi S., Jamshidi S. (2011). September. NTSYSpc 2.02 e implementation in molecular biodata analysis (clustering, screening, and individual selection). Proceedings of 4thInternational Conference on Environmental and Computer Science. Singapore.

[bib38] Kang H., Park W., Mikal S. (2002). Elevated growing temperatures during the day improve the postharvest chilling tolerance of greenhouse-grown cucumber (*Cucumis sativus*) fruit. Postharvest Biol. Technol..

[bib39] Kaushal A., Singh A., Jeena A.S. (2017). Genetic diversity in tomato (*Solanum lycopersicum* L.) genotypes revealed by simple sequence repeats (SSR) markers. J. Appl. Nat. Sci..

[bib40] Khan R.T., Abbas S.R., Gerdezi S.D.A., Javed G., Mehmood A. (2020). 29. Assessment of genetic diversity of tomato genotypes by using molecular markers. Pure Appl. Biol..

[bib41] Khanal B. (2012). Effect of Day and Night Temperature on Pollen Characteristics, Fruit Quality and Storability of Tomato.

[bib42] Kinet J.M., Peet M.M., Wien H.C. (1997). Tomato. The Physiology of Vegetable Crops.

[bib43] Klunklin W., Savage G. (2017). Effect of quality characteristics of tomatoes grown under well-watered and drought stress conditions. Foods.

[bib44] Kumar D., Shukla N., Kumar V., Sahu D.S., Chandel G. (2016). Assessment of genetic variation in tomato (*Solanum lycopersicum* L.) genotypes using SSR molecular markers. Ecol. Environ. Conserv..

[bib45] Kumar S., Prakash P., Srivastava K. (2015). Role of pollen starch and soluble sugar content on fruit set in tomato under heat stress. SABRAO J. Breed. Genet..

[bib46] Kwon Y.S., Park S.G., Yi S.I. (2009). Assessment of genetic variation among commercial tomato (Solanum lycopersicum L) varieties using SSR markers and morphological characteristics. Genes Genomics.

[bib47] Laxman R.H., Srinivasa R.N.K., Bhatt R.M. (2013). Response of tomato (*Lycopersicon esculentum* Mill.) genotypes to elevated temperature. J. Agrometeorol..

[bib48] Levy A., Rabinowitch H.D., Kedar N. (1978). Morphological and physiological characters affecting flower drop and fruit set of tomatoes at high temperatures. Euphytica.

[bib49] Li Z., Palmer W.M., Martin A.P., Wang R., Rainsford F., Jin Y., Patrick J.W., Yang Y., Ruan Y.L. (2012). High invertase activity in tomato reproductive organs correlates with enhanced sucrose import into, and heat tolerance of young fruit. J. Exp. Bot..

[bib50] Lokesha A.N., Shivashankara K.S., Laxman R.H., Geetha G.A., Shankar A.G. (2019). Effect of high temperature on fruit quality parameters of contrasting tomato genotypes. Int. J. Curr. Microbiol. Appl. Sci..

[bib51] Mamatha H., Srinivasa Rao N.K., Laxman R.H., Shivashankara K.S., Bhatt R.M., Pavithra K.C. (2014). Impact of elevated CO_2_ on growth, physiology, yield, and quality of tomato (*Lycopersicon esculentum* Mill) cv. Arka Ashish. Photosynth..

[bib52] Massaretto I.L., Albaladejo I., Purgatto E., Flores F.B., Plasencia F., Egea-Fernández J.M., Bolarin M.C., Egea I. (2018). Recovering tomato landraces to simultaneously improve fruit yield and nutritional quality against salt stress. Front. Plant Sci..

[bib53] Meehl G.A., Washington W.M., Collins W.D., Arblaster J.M., H A., Buja L.E. (2005). How much more global warming and sea level rise. Science.

[bib54] Min L., Li Y., Hu L., Zhu W., Gao Y., Wu Y., Ding S., Liu X., Yang X., Zhang X. (2014). Sugar and auxin signaling pathways respond to high temperature stress during anther development as revealed by transcript profiling analysis in cotton. Plant Physiol..

[bib55] Mitcham E.J., McDonald R.E. (1992). Effect of high temperature on cell wall modifications associated with tomato fruit ripening. Postharvest Biol. Technol..

[bib56] Murray M.G., Thompson W.F. (1980). Rapid isolation of high molecular weight plant DNA. Nucleic Acid Res..

[bib57] Nahar K., Ullah S.M. (2012). Morphological and physiological characters of tomato (*Lycopersicon esculentum* Mill) cultivars under water stress. Bangladesh J. Agric. Res..

[bib58] Pervez M.A., Ayub C.M., Khan H.A., Shahid M.A., Ashraf I. (2009). Effect of drought stress on growth, yield and seed quality of tomato (*Lycopersicon esculentum* L.). Pakistan J. Agric. Sci..

[bib59] Poudyal D., Rosenqvist E.B., Ottosen C. (2019). Phenotyping from lab to field – tomato lines screened for heat stress using *F*v/*F*m maintain high fruit yield during thermal stress in the field. Funct. Plant Biol..

[bib61] Pravallika K., Arunkumar C., Vijayakumar A., Beena R., Jayalekshmi V.G. (2020). Effect of high temperature stress on seed filling and nutritional quality of rice (*Oryza sativa* L.). J. Crop Weed.

[bib62] Punia A., Yadav R., Arora P., Chaudhury A. (2009). Molecular and morphophysiological characterization of superior cluster bean (*Cymopsis tetragonoloba*) varieties. J. Crop. Sci. Biotechnol..

[bib63] Rangana S. (1976). Manual of Analysis of Fruits and Vegetable Products.

[bib65] Root T.L., Price J.T., Hall K.R., Schneider S.H., Rosenzweigk C., Pounds J.A. (2003). Fingerprints of global warming on wild animals and plants. Nature.

[bib66] Rouphael Y., Cardarelli M., Bassal A., Leonardi C., Giuffrida F., Colla G. (2012). Vegetable quality as affected by genetic, agronomic and environmental factors. J. Food Agric. Environ..

[bib67] Sadasivam S., Manickam A. (2008). Biochemical Methods.

[bib68] Sato S., Kamiyama M., Iwata T., Makita N., Furukawa H., Ikeda H. (2006). Moderate increase of mean daily temperature adversely affects fruit set of *Lycopersicon esculentum* by disrupting specific physiological processes in male reproductive development. Ann. Bot***.***.

[bib69] Sato S., Peet M.M., Gardner R.G. (2004). Altered flower retention and developmental patterns in nine tomato cultivars under elevated temperature. Sci. Hortic. (Amst.).

[bib70] Sato S., Peet M.M., Thomas J.F. (2000). Physiological factors limit fruit set of tomato (*Lycopersicon esculentum* Mill.) under chronic, mild heat stress. Plant Cell Environ..

[bib71] Schreiner M., Korn M., Stenger M., Holzgreve L., Altmann M. (2013). Current understanding and use of quality characteristics of horticulture products. Sci. Hortic..

[bib72] Selahle M.K., Sivakumar D., Soundy P. (2014). Effect of photo-selective nettings on post-harvest quality and bioactive compounds in selected tomato cultivars. J. Sci. Food Agric*.*.

[bib73] Shanmugam S., Kjaer K.H., Ottosen C.O., Rosenqvist E., Sharma D.K., Wollenweber B. (2013). The alleviating effect of elevated CO2 on heat stress susceptibility of two wheat (*Triticum aestivum* L.) cultivars. *J. Agron.* Crop Sci..

[bib74] Sharma S.K., Le Maguer M. (1996). Lycopene in tomatoes and tomato pulp fractions. Ital. J. Food Sci..

[bib75] Shehzad T., Okuizumi H., Kawase M., Okuno K. (2009). Development of SSR-based sorghum (*Sorghum bicolor* (L.) Moench) diversity research set of germplasm and its evaluation by morphological traits. Genet. Resour. Crop Evol..

[bib76] Sheoran I.S., Saini H.S. (1996). Drought-induced male sterility in rice: changes in carbohydrate levels and enzyme activities associated with the inhibition of starch accumulation in pollen. Sex. Plant Reprod..

[bib77] Sheshshayee M.S., Parsi Shashidhar G., Madhura J.N., Beena R., Prasad T.G., Udayakumar M., Philippe Monneveux, Jean-Marcel Ribaut (2011). Drought phenotyping in crops: from theory to practice. CGIAR Generation Challenge Programme/CIMMYT.

[bib78] Shi J., Le Maguer M. (2000). Lycopene in tomatoes: chemical and physical properties affected by food processing. Crit. Rev. Food Sci. Nutr..

[bib79] Shivashankara K.S., Pavithra K.C., Laxman R.H., Sadashiva A.T., Roy T.K., Geetha G.A. (2015). Changes in fruit quality and carotenoid profile in tomato (*Solanum lycopersicon* L.) genotypes under elevated temperature. J. Hortic. Sci..

[bib80] Singh R., Singh S., Cheema D.S., Dhaliwal M.S. (2005). Screening for heat tolerance in tomato (*Solanum Lycopersicum* L.). Veg. Sci..

[bib81] Solankey S.S., Akhtar S., Pallavi N., Meenakshi K. (2017). Effect of high temperature stress on morphobiochemical traits of tomato genotypes under polyhouse condition. Indian J. Ecol..

[bib82] Stefan Van Dongen T., Winnepenninckx B. (1996). Multiple UPGMA and neighbor-joining trees and the performance of some computer packages. Mol. Biol. Evol..

[bib83] Tam S.M., Mhiri C., Vogelaar A., Kerkveld M., Pearce S.R., Grandbastien M.A. (2005). Comparative analyses of genetic diversities within tomato and pepper collections detected by retrotransposon-based SSAP, AFLP and SSR. Theor. Appl. Genet*.*.

[bib84] Valverde I.M., Periago M.J., Provan G., Chesson A. (2002). Phenolic compounds, lycopene and antioxidant activity in commercial varieties of tomato (*Lycopersicum esculentum*). J. Sci. Food Agric..

[bib85] Vinocur B., Altman A. (2005). Recent advances in engineering plant tolerance to abiotic stress: achievements and limitations. Curr. Opin. Biotechnol..

[bib86] Vuuren D.P.V., Meinshausenc M., Plattnerd G.K., Joose F., Strassmanne K.M., Smithg S.J. (2008). Temperature increase of 21st century mitigation scenarios. Proc. Natl. Acad. Sci..

[bib87] Wahid A., Gelani S., Ashraf M., Foolad M.R. (2007). Heat tolerance in plants: an overview. Environ. Exp. Bot..

[bib88] Wang S.Y. (2006). Effect of pre-harvest conditions on antioxidant capacity in fruits. Acta Hortic..

[bib89] Wang S.Y., Zheng W. (2001). Effect of plant growth temperature on antioxidant capacity in strawberry. J. Agric. Food Chem..

[bib90] Wang X., Cai X., Xu C., Wang Q., Dai S. (2016). Drought-responsive mechanisms in plant leaves revealed by proteomics. Int. J. Mol. Sci..

[bib91] Wen J., Jiang F., Weng Y., Sun M., Shi X., Zhou Y., Yu L., Wu Z. (2019). Identification of heat-tolerance QTLs and high-temperature stress-responsive genes through conventional QTL mapping, QTL-seq and RNA-seq in tomato. BMC Plant Biol..

[bib92] Xu J., Mieke W., Celestina M., Heidrun H., Ivo R. (2017). Heat stress affects vegetative and reproductive performance and trait correlations in tomato (*Solanum lycopersicum*). Euphytica.

[bib93] Zinn K.E., Tunc-Ozdemir M., Harper J.F. (2010). Temperature stress and plant sexual reproduction: uncovering the weakest links. J. Exp. Bot..

