# Supplementary material for: High temperature induced changes in quality and yield parameters of tomato (Solanum lycopersicum L.) and similarity coefficients among genotypes using SSR markers
Source: Heliyon. 2021 Feb 3;7(2):e05988. doi: 10.1016/j.heliyon.2021.e05988 (PMC7889828; doi:10.1016/j.heliyon.2021.e05988)
Supplement: Gel Pictures [file mmc1.doc]

**Gel profile with DNA bands of tomato**


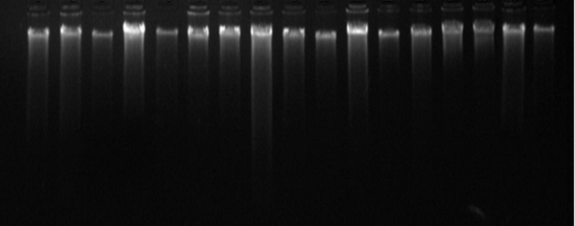


Fig 1(**Gel profile with DNA bands of tomato;** Lane 1-Manuprabha, Lane 2-Akshaya, Lane 3-Pusa Ruby, Lane 4-IC 45, Lane 5- Nandi, Lane 6-IIHR 2200,lane 7-IIHR 26372, lane 8-Palam Pride, lane 9-PKM 1, lane 10-Manulakshmi, lane 11-Arka Sambrat, lane 12-Rakshak, lane 13-Arka Vikas, lane 14-Pusa Rohini, lane 15-Arka alok , lane 16-Sakthi, lane 17-Vaibhav)


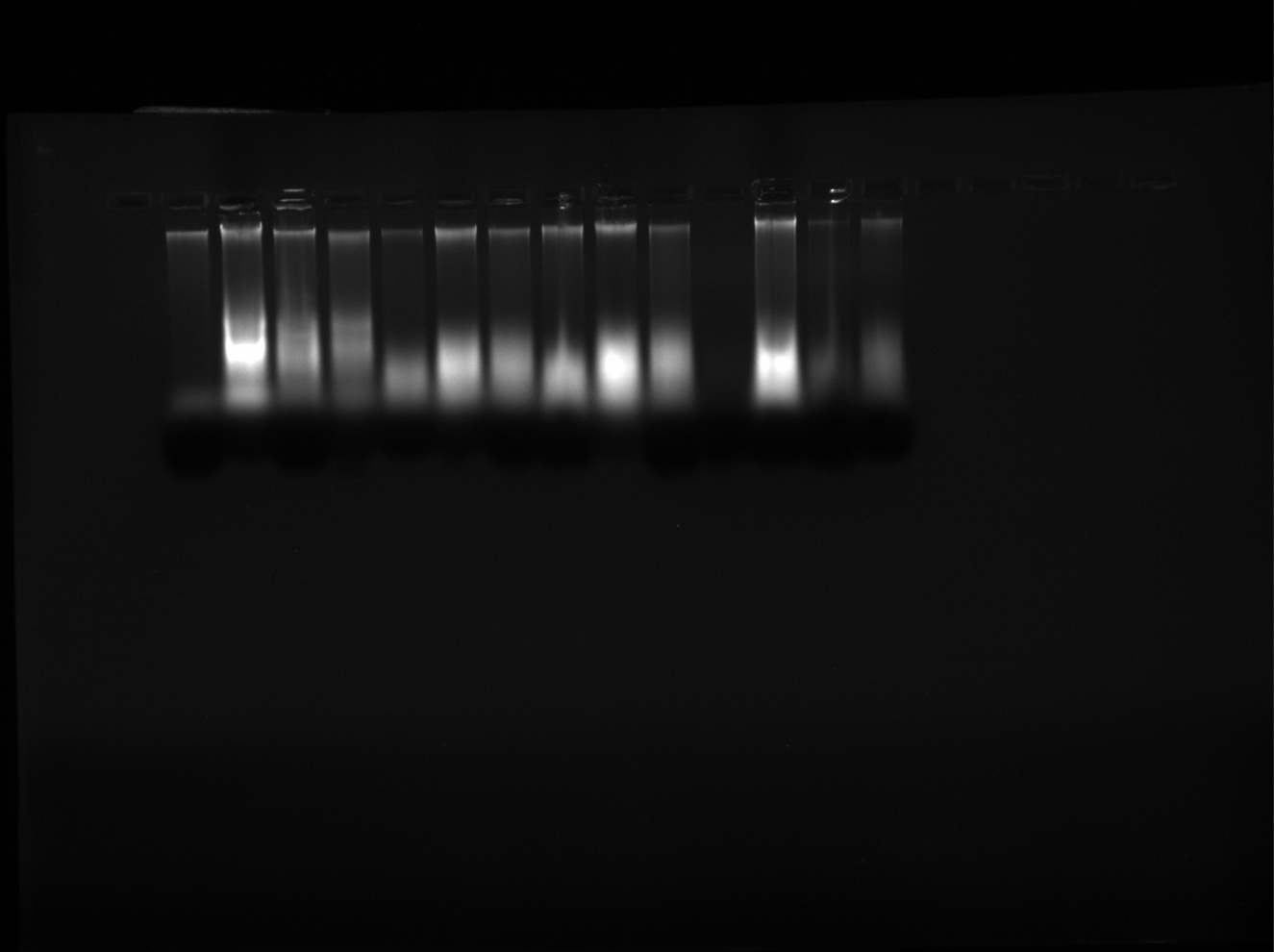


**Fig 1:Gel profile with DNA bands of tomato**: ( Lane 1-IIHR 2200, lane 2- Vellayani Vijay, lane 3-IIHR 26372, lane 4-Palam Pride, lane 5-PKM 1, lane 6-Manulakshmi,lane 7-Anagha, lane 8-Kashi Vishesh, lane 9- Arka Sourabh, lane 10-Arka Sambrat, lane 11-Rakshak, lanelane 12-Arka Abha).

**Amplification pattern of 22 tomato varieties obtained by SSR marker SSR 96**


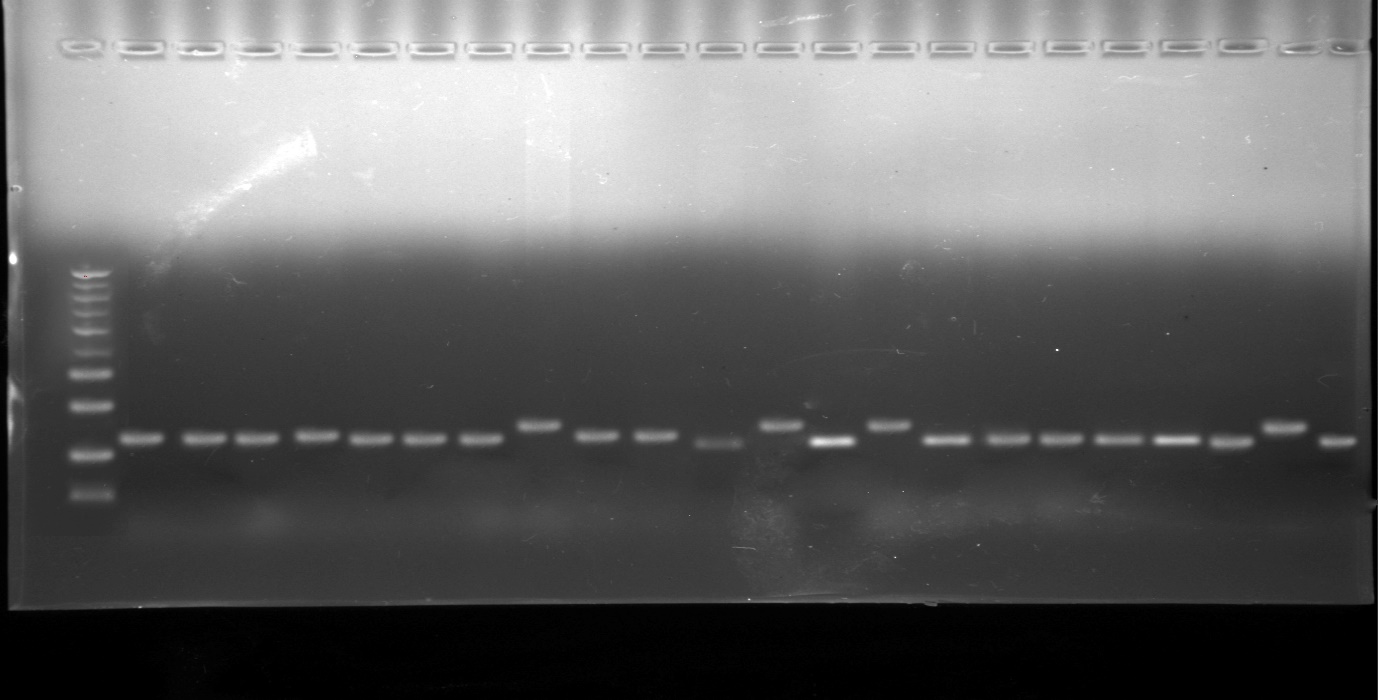


SSR 96

**1 2 3 4 5 6 7 8 9 10 11 12 13 14 15 16 17 18 19 20 21 22 23**

200bp

10010

100bp

10010

(Fig 7. Amplification pattern of 22 tomato genotypes obtained by SSR marker SSR 96. Lane 1- 100bp ladder, Lane 2-Manuprabha, Lane 3-Akshaya, Lane 4-Pusa Ruby, Lane 5-IC-45, Lane 6- Nandi, Lane 7-IIHR-2200, lane 8-IIHR-26372, lane 9-Palam Pride, lane 10-PKM-1, lane 11-Manulakshmi, lane 12-Arka Samrat, lane 13- Arka Rakshak, lane 14-Arka Vikas, lane 15-Pusa Rohini, lane 16-Arka Alok , lane 17-Sakthi, lane 18-Vaibhav, lane 19- Vellayani Vijay, lane 20-Anagha, lane 21-Kashi Vishesh, lane 22- Arka Saurabh, lane 23-Arka Abha).

**Amplification pattern of 22 tomato varieties obtained by SSR marker SSR 63**


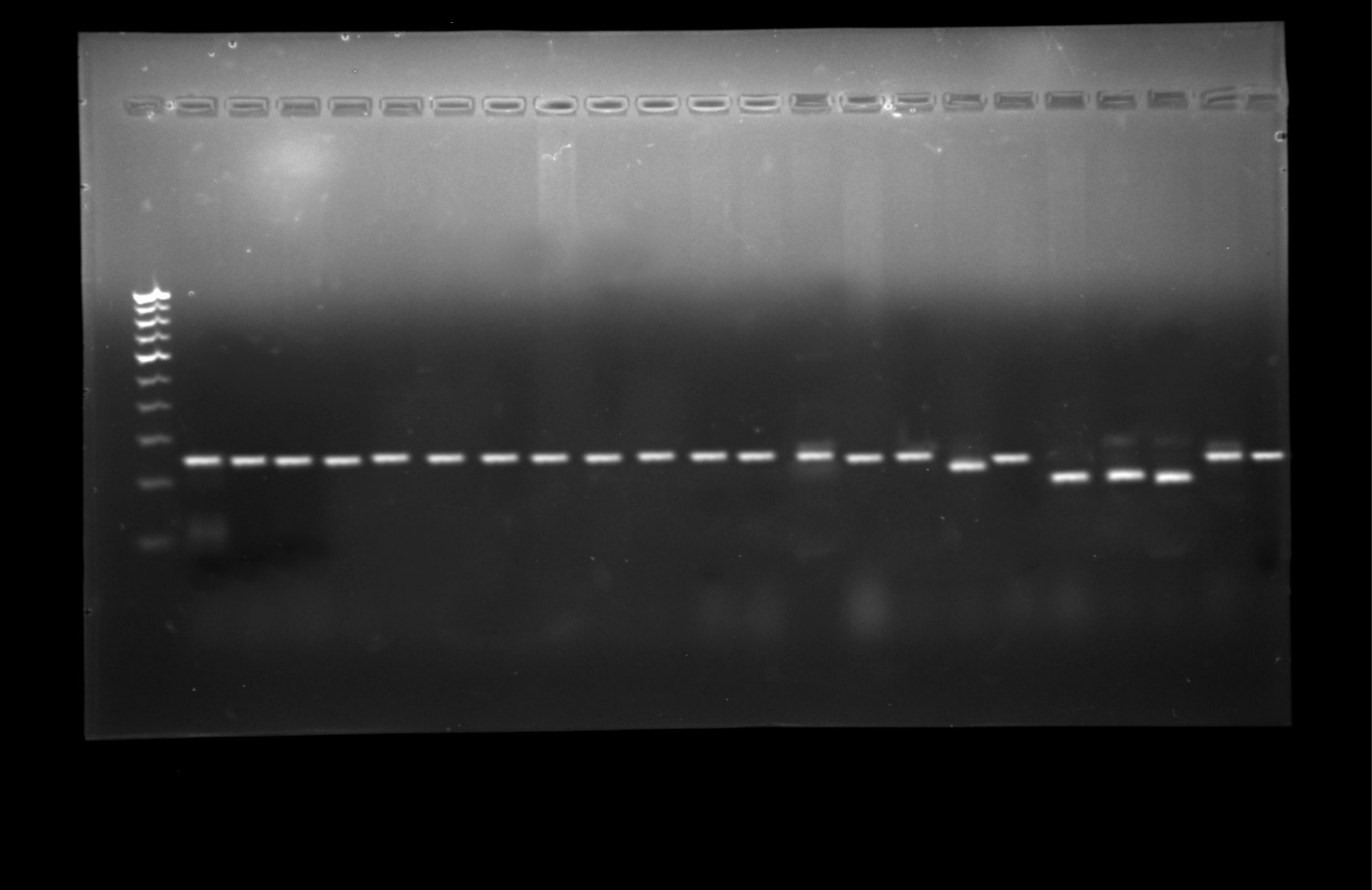


SSR63

**1 2 3 4 5 6 7 8 9 10 11 12 13 14 15 16 17 18 19 20 21 22 23**

200bp

10010

100bp

10010

(Fig 7: Amplification pattern of 22 tomato genotypes obtained by SSR marker SSR 63.Lane 1- 100bp ladder, Lane 2-Manuprabha, Lane 3-Akshaya, Lane 4-Pusa Ruby, Lane 5-IC-45, Lane 6- Nandi, Lane 7-IIHR-2200, lane 8-IIHR-26372, lane 9-Palam Pride, lane 10-PKM-1, lane 11-Manulakshmi, lane 12-Arka Samrat, lane 13- Arka Rakshak, lane 14-Arka Vikas, lane 15-Pusa Rohini, lane 16-Arka Alok , lane 17-Sakthi, lane 18-Vaibhav, lane 19- Vellayani Vijay, lane 20-Anagha, lane 21-Kashi Vishesh, lane 22- Arka Saurabh, lane 23-Arka Abha).

**Amplification pattern of 22 tomato varieties obtained by SSR marker SSR 13**


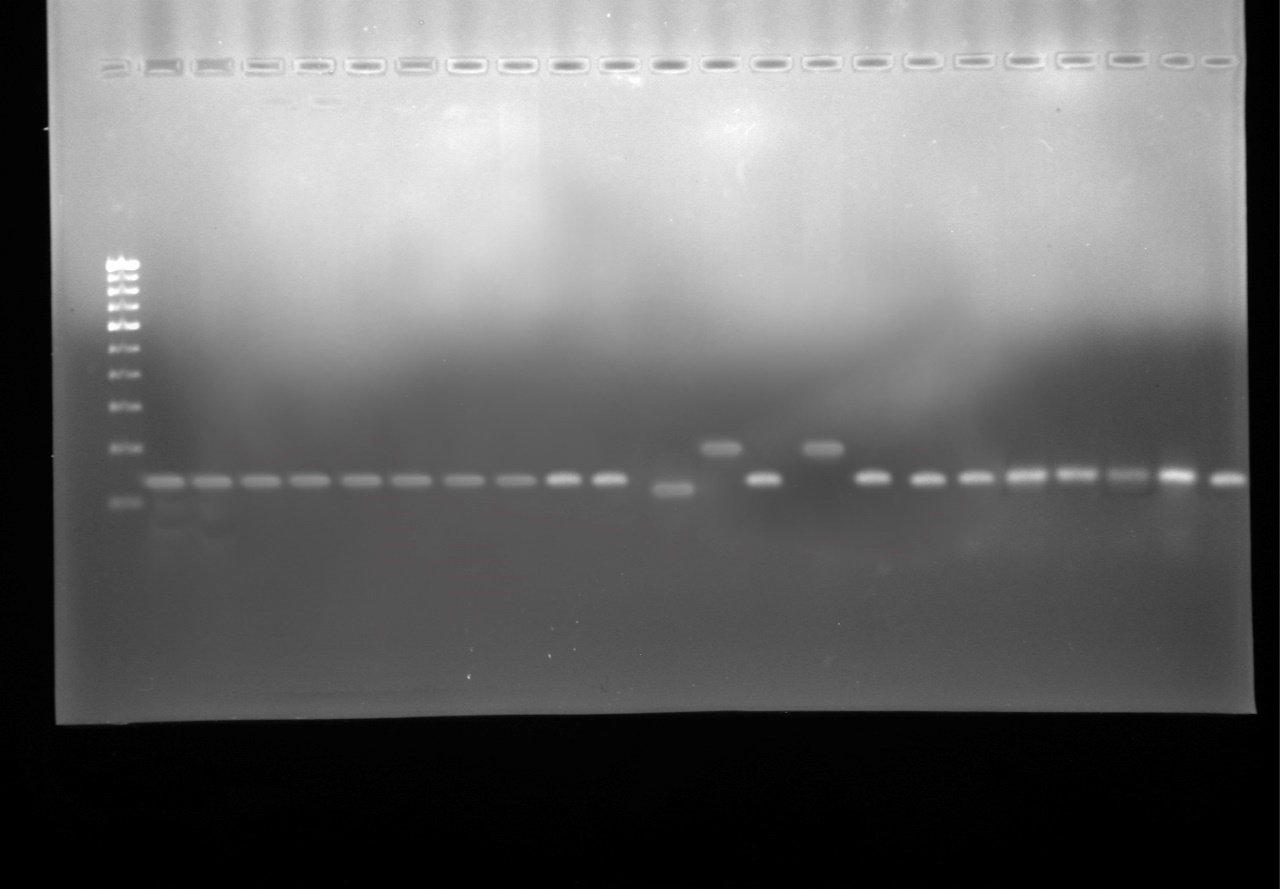


SSR13

**1 2 3 4 5 6 7 8 9 10 11 12 13 14 15 16 17 18 19 20 21 22 23**

200bp

10010

100bp

10010

(Fig: 7. Amplification pattern of 22 tomato genotypes obtained by SSR marker SSR 13. Lane 1- 100bp ladder, Lane 2-Manuprabha, Lane 3-Akshaya, Lane 4-Pusa Ruby, Lane 5-IC-45, Lane 6- Nandi, Lane 7-IIHR-2200, lane 8-IIHR-26372, lane 9-Palam Pride, lane 10-PKM-1, lane 11-Manulakshmi, lane 12-Arka Samrat, lane 13- Arka Rakshak, lane 14-Arka Vikas, lane 15-Pusa Rohini, lane 16-Arka Alok , lane 17-Sakthi, lane 18-Vaibhav, lane 19- Vellayani Vijay, lane 20-Anagha, lane 21-Kashi Vishesh, lane 22- Arka Saurabh, lane 23-Arka Abha).

**Amplification pattern of 22 tomato varieties obtained by SSR marker SSR 270**


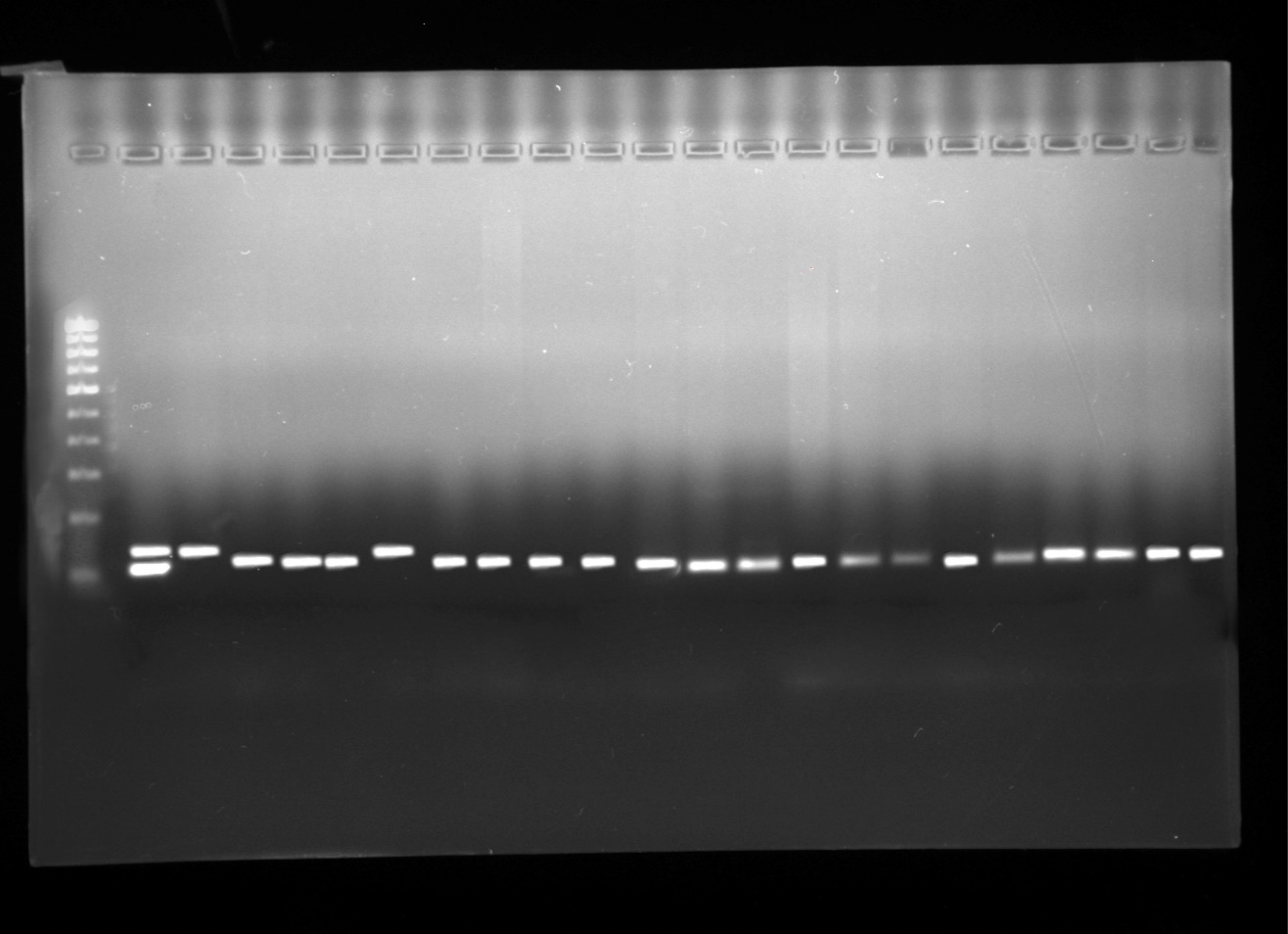


SSR270

**1 2 3 4 5 6 7 8 9 10 11 12 13 14 15 16 17 18 19 20 21 22 23**

200bp

10010

100bp

10010

(Fig 7:. Amplification pattern of 22 tomato genotypes obtained by SSR marker SSR 270. Lane 1- 100bp ladder, Lane 2-Manuprabha, Lane 3-Akshaya, Lane 4-Pusa Ruby, Lane 5-IC-45, Lane 6- Nandi, Lane 7-IIHR-2200, lane 8-IIHR-26372, lane 9-Palam Pride, lane 10-PKM-1, lane 11-Manulakshmi, lane 12-Arka Samrat, lane 13- Arka Rakshak, lane 14-Arka Vikas, lane 15-Pusa Rohini, lane 16-Arka Alok , lane 17-Sakthi, lane 18-Vaibhav, lane 19- Vellayani Vijay, lane 20-Anagha, lane 21-Kashi Vishesh, lane 22- Arka Saurabh, lane 23-Arka Abha).

**Amplification pattern of 22 tomato varieties obtained by SSR marker SSR 356**


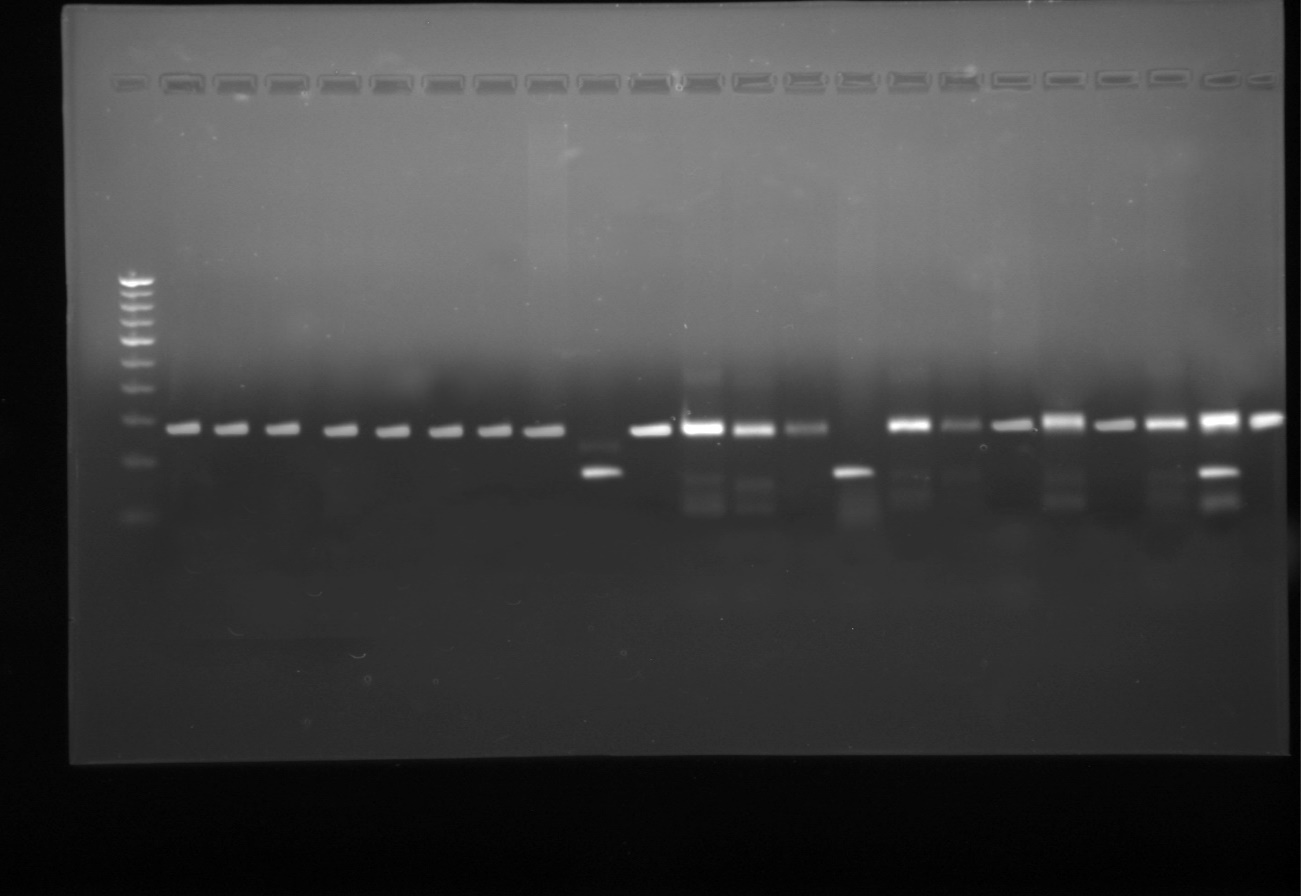


SSR356

**1 2 3 4 5 6 7 8 9 10 11 12 13 14 15 16 17 18 19 20 21 22 23**

200bp

10010

100bp

10010

(Fig 7. Amplification pattern of 22 tomato genotypes obtained by SSR marker SSR 356. Lane 1- 100bp ladder, Lane 2-Manuprabha, Lane 3-Akshaya, Lane 4-Pusa Ruby, Lane 5-IC-45, Lane 6- Nandi, Lane 7-IIHR-2200, lane 8-IIHR-26372, lane 9-Palam Pride, lane 10-PKM-1, lane 11-Manulakshmi, lane 12-Arka Samrat, lane 13- Arka Rakshak, lane 14-Arka Vikas, lane 15-Pusa Rohini, lane 16-Arka Alok , lane 17-Sakthi, lane 18-Vaibhav, lane 19- Vellayani Vijay, lane 20-Anagha, lane 21-Kashi Vishesh, lane 22- Arka Saurabh, lane 23-Arka Abha).

**Amplification pattern of 22 tomato varieties obtained by SSR marker SSR 605**


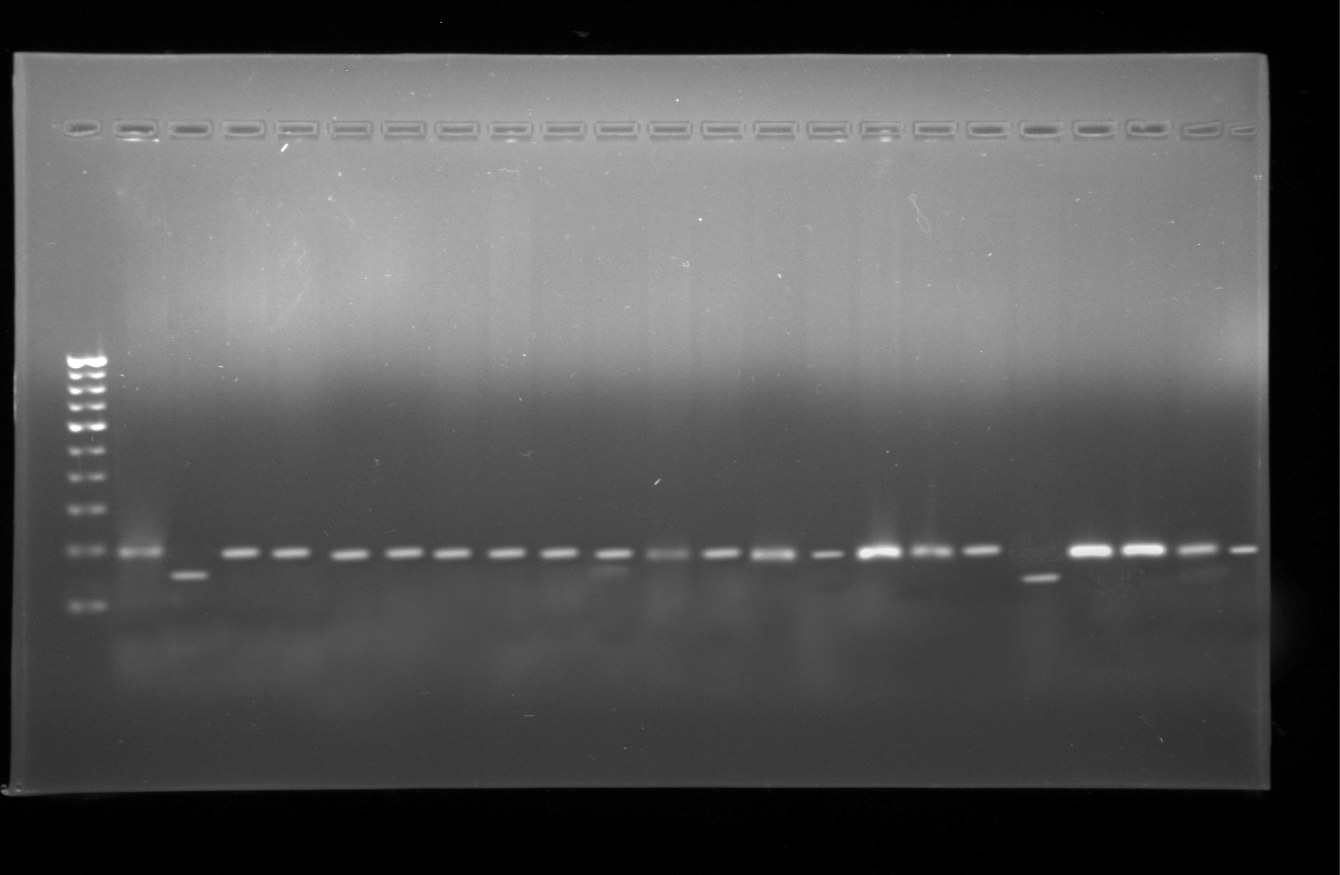


SSR605

**1 2 3 4 5 6 7 8 9 10 11 12 13 14 15 16 17 18 19 20 21 22 23**

200bp

10010

100bp

10010

**(**Fig 7. Amplification pattern of 22 tomato genotypes obtained by SSR marker SSR 605. Lane 1- 100bp ladder, Lane 2-Manuprabha, Lane 3-Akshaya, Lane 4- Pusa Ruby, Lane 5-IC-45, Lane 6- Nandi, Lane 7-IIHR-2200, lane 8-IIHR-26372, lane 9-Palam Pride, lane 10-PKM-1, lane 11-Manulakshmi, lane 12-Arka Samrat, lane 13- Arka Rakshak, lane 14-Arka Vikas, lane 15-Pusa Rohini, lane 16-Arka Alok , lane 17-Sakthi, lane 18-Vaibhav, lane 19- Vellayani Vijay, lane 20-Anagha, lane 21-Kashi Vishesh, lane 22- Arka Saurabh, lane 23-Arka Abha).

**Amplification pattern of 22 tomato varieties obtained by SSR marker SSR 248**


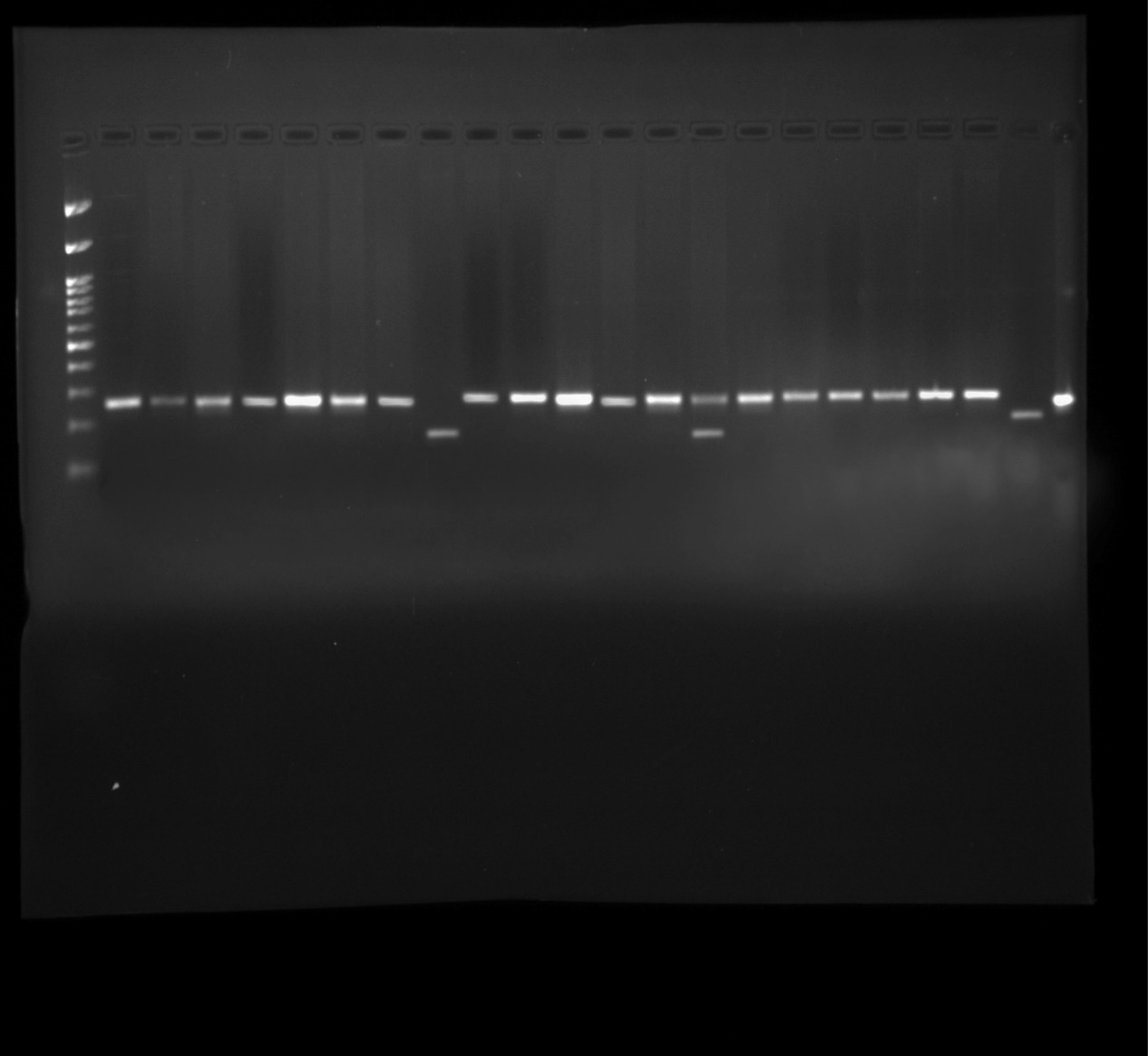


SSR248

**1 2 3 4 5 6 7 8 9 10 11 12 13 14 15 16 17 18 19 20 21 22 23**

200bp

100bp

(Fig 7: . Amplification pattern of 22 tomato genotypes obtained by SSR marker SSR 248. Lane 1- 100bp ladder, Lane 2-Manuprabha, Lane 3-Akshaya, Lane 4-Pusa Ruby, Lane 5-IC-45, Lane 6- Nandi, Lane 7-IIHR-2200, lane 8-IIHR-26372, lane 9-Palam Pride, lane 10-PKM-1, lane 11-Manulakshmi, lane 12-Arka Samrat, lane 13- Arka Rakshak, lane 14-Arka Vikas, lane 15-Pusa Rohini, lane 16-Arka Alok , lane 17-Sakthi, lane 18-Vaibhav, lane 19- Vellayani Vijay, lane 20-Anagha, lane 21-Kashi Vishesh, lane 22- Arka Saurabh, lane 23-Arka Abha).

(
